# Vegan versus meat-based dog food: Guardian-reported health outcomes in 2,536 dogs, after controlling for canine demographic factors

**DOI:** 10.1016/j.heliyon.2024.e35578

**Published:** 2024-08-05

**Authors:** Andrew Knight, Alexander Bauer, Hazel J. Brown

**Affiliations:** a School of Veterinary Medicine, College of Environmental and Life Sciences, Murdoch University, 90 South St., Murdoch, WA 6150, Australia; b School of Environment and Science, Nathan Campus, Griffith University, 170 Kessels Rd, Nathan, QLD, 4111, Australia; c Faculty of Health and Wellbeing, University of Winchester, Sparkford Road, Winchester, SO22 4NR, UK; d Statistical Consulting Unit StaBLab, Department of Statistics, LMU Munich, Ludwigstr. 33, 80539, Munich, Germany

**Keywords:** Dog, Canine, *Canis familiaris*, Pet food, Dog food, Diet, Raw meat, Vegan

## Abstract

To compare health outcomes between dogs fed meat and vegan diets, we surveyed 2,536 dog guardians who provided data and opinions about dogs fed conventional meat (1,370 = 54 %), raw meat (830 = 33 %) or vegan (336 = 13 %) diets for at least one year. We examined seven general indicators of illness: increased numbers of veterinary visits, medication use, progression onto a therapeutic diet after initial maintenance on a vegan or meat-based diet, reported veterinary assessment of being unwell, reported veterinary assessment and guardian opinion of increased illness severity, and number of health disorders per unwell dog. We also considered the prevalence of 22 specific health disorders, based on reported veterinary assessments. In each dietary group the proportions of dogs considered to have suffered from health disorders were: conventional meat – 49 %, raw meat – 43 % and vegan – 36 %. Probabilities of suffering from a disorder respectively appeared highest in dogs fed conventional meat for 11 disorders, raw meat for eight disorders, and vegan diets for three disorders. We conducted regression analyses to control for differences in medically-relevant canine demographic variables, including age, sex, neutering status, breed size and unusually high exercise levels. Dogs fed vegan diets had the best health outcomes. This trend was clear and consistent, with dogs fed vegan diets usually having substantial and statistically significant decreases in risks of these seven general indicators of illness. These ranged from 14.4 % to 51.3 % compared to dogs fed conventional meat-based diets. For six specific disorders, vegan diets were associated with statistically significant risk reductions of 50 %–61 % compared to dogs fed conventional meat. After pooling our results with related studies published to date, vegan dog food was consistently associated with lowered risks of multiple specific health disorders. No health disorder was consistently more prevalent in dogs fed vegan diets.

## Highlights

• Dogs fed vegan diets had the best health outcomes. This trend was clear and consistent.
• Health disorder rates: conventional meat – 49 %, raw meat – 43 %, vegan – 36 %.
• Illness indicators were 14.4 %–51.3 % less common among vegans vs. conventional meat.
• Six specific disorders had 50 %–61 % lower odds among vegans vs. conventional meat.
• Vegan dog food is consistently associated with lowered risks of certain health disorders.

## Graphical abstract

**Figure:**
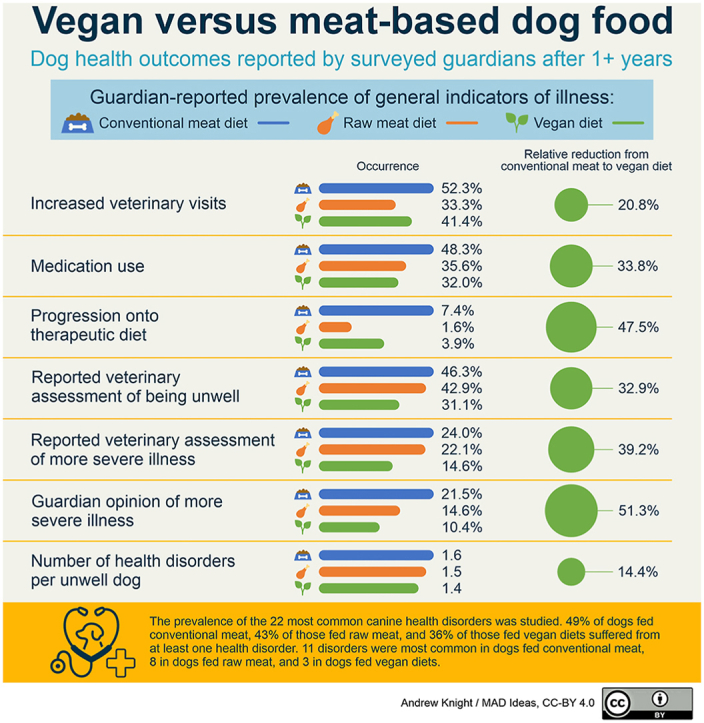

## 1 Introduction

Meat-based pet foods can have adverse impacts for environmental sustainability and farmed animal welfare [[1], [2], [3], [4], [5]]. Increasing concerns about such aspects is driving the development of alternatives including *in vitro* meat products, and pet foods based on other novel protein sources, including terrestrial plants, insects, yeast, fungi and seaweed. Of these, the vegan pet food market is most developed. It was valued at USD 8.7 billion globally by 2020, and forecast to be worth USD 15.7 billion by 2028 – a compound annual growth rate of 7.7 % [6].

However, as noted in our previous study in this series [7], some are concerned that vegan diets may be harmful to dogs and cats. Loeb [8] claimed there were “… almost insurmountable challenges – biological, legal and downright practical – facing anyone attempting to shoehorn dogs and cats into a vegan dietary system” and concluded “… it's fairly clear that feeding a dog a vegan diet is not recommended.”

There are two clear routes for assessing the nutritional suitability of vegan diets for dogs. One is to examine in detail the steps taken by pet food manufacturers to ensure the quality and nutritional soundness of their products. This was recently performed within a survey of 29 companies producing meat-based (19) and plant-based (10) pet foods [9]. Although potential for limited improvements existed, most manufacturers were assessed as having acceptable or superior standards, at nearly all stages throughout the design, manufacturing, transportation and storage of pet foods. Standards used for plant-based diets were slightly superior to those utilized for meat-based diets.

Arguably the ultimate test of nutritional suitability, however, is the animal health outcomes [7]. Feeding trials are consequently considered the gold standard method for assessing the nutritional soundness of new formulations [10,11]. The effects of plant-based diets on the health status of dogs have been previously studied and reviewed [12]. However, the samples included within most of those studies have historically been small, limiting their predictivity for dog populations. By 2021, no published large-scale study of dogs had examined how health indicators varied between dogs maintained on vegan or meat-based diets. Accordingly, in 2020 we surveyed 2,639 dog guardians, asking about one dog living with them, for at least one year. 2,536 provided information relating to a single dog, fed a conventional meat (1,370 = 54 %), raw meat (830 = 33 %) or vegan (336 = 13 %) diet [7]. Dogs fed conventional diets appeared to fare worse than those fed either of the other two diets. Dogs fed raw meat initially appeared to fare marginally better than those fed vegan diets. However, there were sizeable and statistically significant differences in average ages. Dogs fed raw meat were younger, which has been demonstrated to be associated with improved health outcomes.

Accordingly, we designed a further study, to assess and control for differences between dietary groups in medically-relevant canine demographic variables, notably: age, sex, neutering status, breed size and exercise levels. Our null hypothesis was that guardian-reported canine health indicators would not significantly vary with diet type, after controlling for these medically-relevant canine demographic factors. Results of other survey parts were recently reported (palatability of different diets [13], health outcomes in cats [14]), or are the subject of related, forthcoming studies.

## 2 Materials and methods

Some details of our methodology were described previously [7]. Relevant parts are summarised here for completeness. A survey for dog or cat guardians was designed using the ‘Online surveys’ platform (https://www.onlinesurveys.ac.uk). Respondents were asked about themselves and one dog or cat living with them for at least a year. They were asked to specify whether their pet's diet was based on conventional, raw or *in vitro* meat, insects, fungi or algae, or whether it was a vegetarian, vegan or another diet. They were asked to consider the main ingredients within their pet's normal diets, and only one diet type could be selected. Vegetarian diets were defined as excluding meat, but including eggs or milk, and vegan diets as excluding all animal products. Information was also obtained about the use of treats, snacks, scraps or supplements. Although the original survey inquired about both dogs and cats, this current study analysed only the canine outcomes. A similar study focusing on the feline outcomes was recently published [14].

Both human and animal demographic information was elicited. For humans, this included location (continental region, urban or rural), educational qualifications, occupation, household income, age, gender and personal diet. For animals this included role (companion or working animal), age, sex/neuter status, exercise level and health status. Breed sizes were also determined for dogs, and were defined in the survey as: toy: ≤4.1 kg or ≤9 lb, small: 4.5–10.9 kg or 10–24 lb, medium: 11.4–22.3 kg or 25–49 lb, large: 22.7–40.5 kg or 50–89 lb, and giant: >40.9 kg or >90 lb [15].

The survey inquired about general indicators of illness (1–7 following), and about the presence of specific health disorders based on reported assessments by veterinarians, during the previous year. If prescription or therapeutic diets were currently in use, guardians were asked to base answers on the diet used during the year prior to the commencement of the therapeutic diet. The seven general indicators of illness included (1) the frequency of veterinary visits, and (2) of medication use (excluding routine vaccinations, parasite treatments, treatments associated with neutering operations, or microchipping). Guardians were also asked (3) whether their dog had progressed onto a therapeutic diet, after being initially maintained on another type of diet. Guardians were asked to provide (4) their own opinion about their dog's health status, and also to report (5) their veterinarian's assessment. Possible answers ranged from no problems/routine preventative healthcare, up to seriously ill. Where veterinarians reportedly considered dogs to be unwell, guardians were asked which disorder(s) were present, within 18 disorders reportedly among the most common among dogs [[16], [17], [18], [19], [20]]. Multiple disorders could be selected, and details of additional disorders could be provided by selecting ‘other’. Such ‘other’ disorders were examined, and reclassified into the 18 existing or four new disorder types, yielding 22 possible health disorders in total.

When analysing health disorders, cases were only included where veterinary visits had occurred at least once in the previous year, and where guardians were reportedly sure of the assessments of their veterinarians. This subset was used to calculate the prevalence of the 22 specific health disorders, (6) the percentage of unwell dogs, and (7) the average number of cases of health disorder, per unwell dog.

### 2.1 Potentially confounding factors

We were primarily interested in possible effects of main diets fed, on dogs within normal households. Treats may deviate from main diets, in ingredients and proportions. However, we chose not to exclude from consideration dogs who received regular treats, predicting most dogs would fall within this group. Health status may be affected by animal demographic factors such as age, sex, desexing (neutering) status, breed and exercise level [[19], [20], [21], [22]]. Hence, we sought to ascertain differences in these factors between the dietary groups we studied, and to control for differences via regression analyses (see Statistical analysis, following). We chose not to explore certain additional demographic factors. For example, the prevalence of certain diseases varies with specific canine breed [21,22]. However, small numbers within breed groups were expected to limit our ability to statistically analyse any apparent differences, hence we elected not to discriminate by specific breed. Numbers within breed size groups (which comprise multiple breeds of similar sizes) were larger, and so we did examine differences in breed sizes, between dietary groups. Similarly, exercise levels may affect health outcomes, and these vary widely amongst domesticated dogs. We generally did not attempt to differentiate between exercise levels, although we did explore differences between dietary groups in unusually high exercise levels – as exemplified by racing greyhounds, working farm dogs, or police dogs – which usually differ from those of dogs within normal households.

### 2.2 Survey pilot and distribution

We used the ‘Online surveys’ platform to host our survey. This complied with the UK General Data Protection Regulation, and by 2019 was used by 88 % of UK higher education institutions [23], including our University of Winchester.

**Fig. A1:**
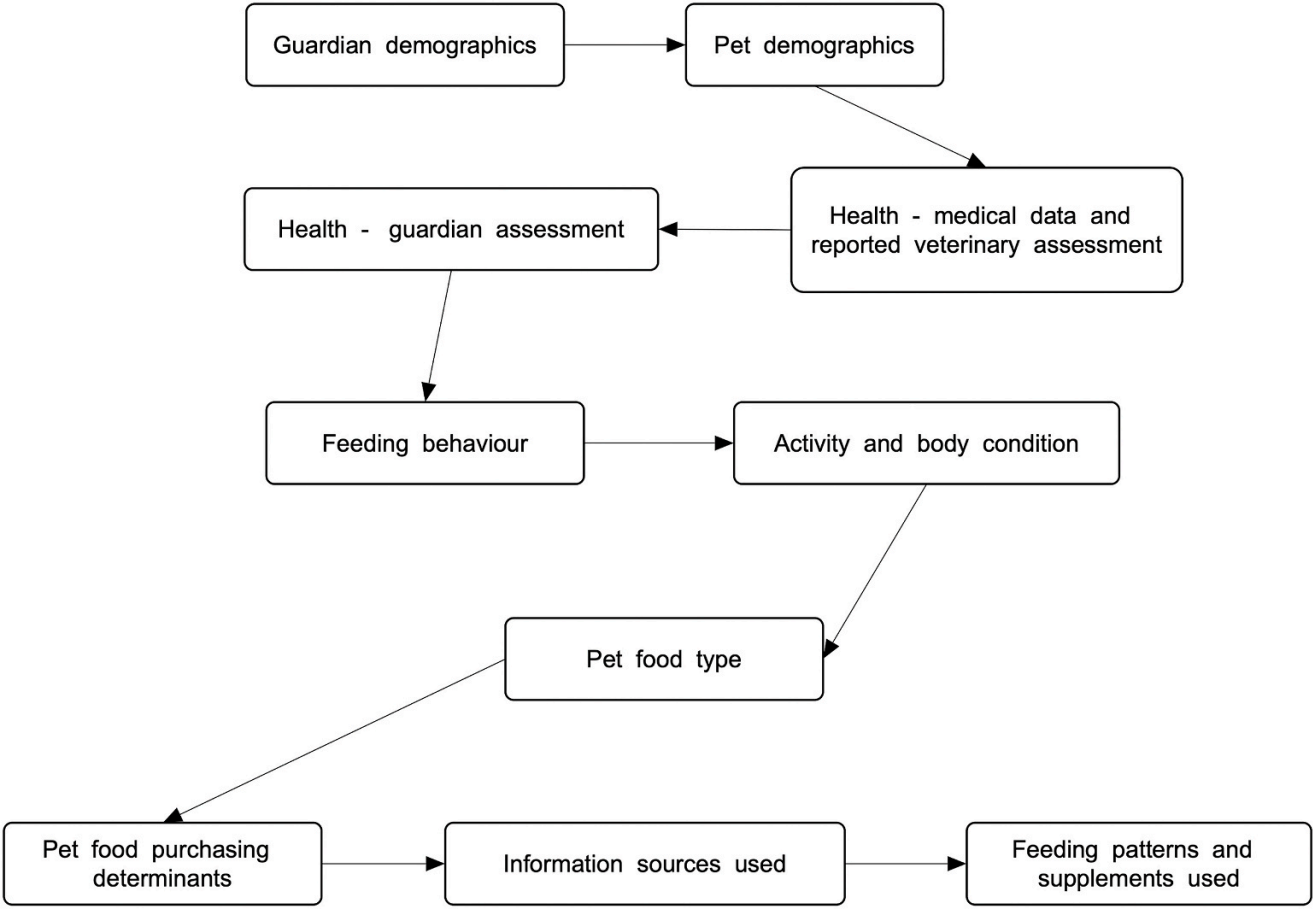
Survey parts [7].

In April 2020 we piloted our survey to 25 respondents. Based on feedback improvements were made to survey questions and structure. Realizing that a guardian reporting use of an unconventional diet might consciously or unconsciously underestimate any health problems in subsequent survey answers, we repositioned questions about health toward the beginning of the survey. Similarly, questions about veterinary opinions of animal health were repositioned toward the beginning. We aimed to reposition variables likely to be dependent, prior to any possibly corresponding independent variables. Some questions were also clarified and simplified. The final survey steps are shown in Figure A1.

The final survey was open from May–December 2020. To maximize exposure the survey was advertised to dog and cat interest groups within social media, using both paid Facebook advertising and volunteers. Facebook advertising terms relating to dogs and cats were chosen, but were not otherwise limited. Expecting lower levels of unconventional diet use, and considering the need to achieve sample sizes sufficient for statistical analysis, efforts were also made to reach unconventional pet food interest groups. However, no bias towards or against any particular diets were explicit or implied within advertising materials, survey questions or explanatory text.

### 2.3 Statistical analysis

After initial examination of diets, subsequent analysis was limited to dogs maintained on the three largest diet groups: conventional meat-based, raw meat-based and vegan pet food. Initially, we examined the association of these three main diet types with canine demographic factors. Association with the categorical variables joint ‘sex and neuter status’, each of ‘sex’ and ‘neuter status’ individually, and ‘breed size’ and ‘exercise level’, were all investigated using chi-square tests of independence [24]. For variables that showed significant differences, we provided effect size interpretations using the Cramer's V statistic, with small, medium or large effects interpreted when V was close to 0.2, 0.5 and 0.8, respectively [25]. Differences between mean ages were explored using an ANOVA (analysis of variance) test, and – as this test showed significant differences, also using three pairwise independent samples t-tests with unequal variances (“Welch test”) [24]. When pairwise significant differences in these mean ages were detected, effect size interpretations were provided using the Cohen's d statistic, with small, medium and large effects interpreted when |d| was close to 0.2, 0.5 and 0.8, respectively [26].

Next, associations were investigated between the three main diet types and dog health. As noted, guardians provided information about seven general indicators of illness, and about the prevalence of 22 specific health disorders. The general indicators of illness were: increased numbers of veterinary visits, medication use, progression onto a therapeutic diet after initial maintenance on a vegan or meat-based diet, reported veterinary assessment of being unwell, reported veterinary assessment and guardian opinion of more severe illness, and number of health disorders per unwell dog. The 22 specific disorders reflected veterinary assessments as reported by guardians.

Potential associations of the diet type with all seven indicators of illness were investigated using separate generalized additive regression models (GAMs; [27]). The estimation of additive regression has two main benefits compared to individual hypothesis tests. First, regression models allow adjustment for differences between the dietary groups in control variables, e.g., controlling for the fact that the dogs in our study fed raw meat were, on average, 1.8 years younger than dogs fed a vegan diet. Second, additive regression allows for the estimation of nonlinear effects. In our models, the effect of age was estimated nonlinearly (based on a P-spline basis with four basis functions). This is necessary since, e.g., the number of veterinary visits does not rise or decline by a constant factor every year, but follows a ‘U’ shape where very young and very old dogs both have higher number of veterinary visits, independent of diet. All seven regression models included the same set of control variables: age, sex and neuter status, breed size, and a binary indicator denoting unusually high exercise levels. For the categorical variables, we assigned the categories occurring most frequently as the reference categories.

Depending on the variable type for each health indicator, different types of regression models were estimated (see Ref. [28] for all following types). Binary logistic regression was estimated for variables ‘increased numbers of veterinary visits (comparing ‘0–1 visits’ to ‘2 or more visits’), ‘medication use’, ‘progression onto a therapeutic diet’ and ‘reported veterinary assessment of being unwell’. Ordinal logistic regression was estimated for variables ‘reported veterinary assessment of more severe illness' and ‘guardian opinion of more severe illness' (comparing the two consecutive thresholds between the three categories ‘healthy’, ‘minor problems’ and ‘frequent problems or seriously ill’). Quasi-Poisson regression was estimated for the variable ‘number of health disorders per unwell dog’, with an estimated dispersion parameter of 0.52.

Consistent with state-of-the-art statistical practice, our interpretations followed the American Statistical Association's statement on the use of p-values [29,30]. Accordingly, we used p-values and confidence intervals with a significance level of 0.05 to evaluate the (un)certainty of the effects, but not as an evaluation of the “relevance” of effect sizes. Instead, the main focus lies on effect strength, measured based on the coefficient value rather than the p-value. Results were interpreted as “association” (when a significant effect ∼10 % or stronger), “strong tendency” (when a non-significant effect ∼25 % or stronger), “tendency” (when a non-significant effect ∼10 % or stronger) or “marginal” (when a non-significant effect <10 %). In light of this, and given the number of comparisons associated with comparing three dietary groups, we chose not to correct p-values for multiple testing.

As well as exploring associations between dietary differences and the seven general health indicators, associations with the 22 specific health disorders were also explored. For each disorder, a separate additive logistic regression model was estimated, including age (nonlinear), sex and neuter status, breed size, and exercise level, as linear control variables.

Goodness of fit was evaluated using area under the curve (AUC) values for (ordinal) logistic regression, and the explained share of deviance and the mean absolute error (MAE) for the Quasi-Poisson regression [28]. AUC and MAE values were calculated on a randomly drawn 20 % test set, based on a re-estimation of the regression models on the respective 80 % training set. AUC values were 0.61 (variable ‘increased numbers of veterinary visits’), 0.62 (‘medication use’), 0.70 (‘progression onto a therapeutic diet’), 0.63 (‘reported veterinary assessment of being unwell’), 0.60 and 0.67 (for the two thresholds for variable ‘reported veterinary assessment of more severe illness’), and 0.71 and 0.68 (for the two thresholds for variable ‘guardian opinion of more severe illness’). All AUC values indicate an acceptable or good fit. The results of the Quasi-Poisson model for variable ‘number of health disorders per unwell dog’ should be interpreted with some care, with the model having an 11 % explained share of deviance and a MAE of 1.29.

All interpretations were based on a significance level of 0.05. All analyses were conducted with the open-source software R [31]. Regression model estimation was performed using function *gam* from R package *mgcv* [27].

## 3 Results

Our initial results (prior to controlling for canine demographic factors) were previously reported [7]. Some are reproduced here for completeness.

### 3.1 Canine demographic factors

#### 3.1.1 Diets

**Fig. 1:**
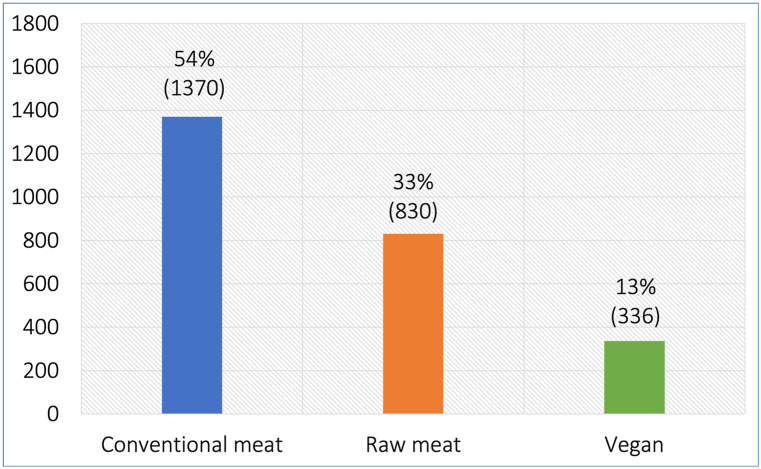
Three main diets fed to 2,536 dogs. After Knight et al. [7].

Of the 2,536 dogs included in this study, 1,370 (54 %) were maintained on conventional meat-based diets, 830 (33 %) on raw meat-based diets, and 336 (13 %) on vegan diets (Fig. 1). A total of 1,935 (76 %) received treats/snacks/scraps at least once daily, and 926 (37 %) received regular dietary supplements [7].

#### 3.1.2 Ages

**Fig. 2:**
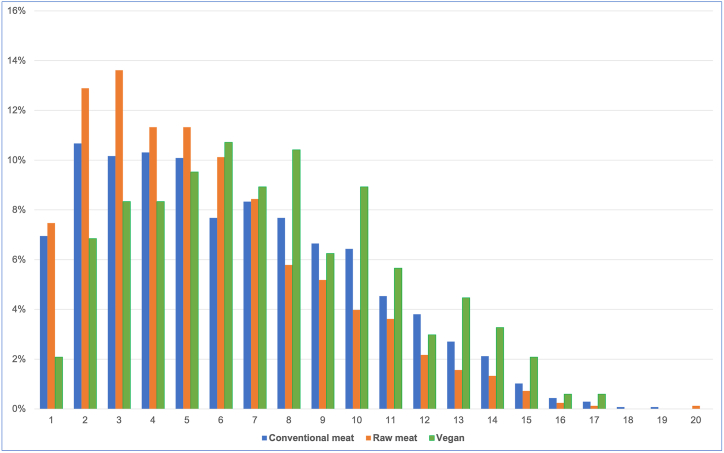
Ages of 2,534 dogs fed three main diets [7].

Guardians were unsure of dogs’ ages in two cases. Ages of the remaining 2,534 dogs are shown in Fig. 2. The mean ages were (in years): overall – 6.18, raw meat – 5.52, conventional meat – 6.31, vegan – 7.30. Differences between the groups were all significant, and of small to medium size (Table A1).

#### 3.1.3 Sex/neuter status

**Fig. 3:**
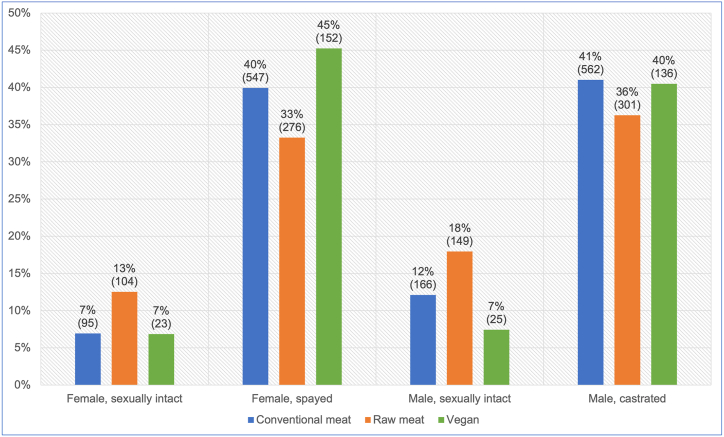
Sex/neuter status of 2,536 dogs fed three main diets. After Knight et al. [7].

The sex/neuter status of these 2,536 dogs is provided in Fig. 3. Females and males comprised 47 % and 53 % respectively. A chi-square test of independence showed diet to be significantly associated with sex/neuter status (p < 0.0001). However, the effect size was small (Cramer's V = 0.106). Dogs fed vegan diets were slightly more likely to be female within this sample, but sex and diet type were not statistically significantly associated (p = 0.139) [7].

With respect to desexing however, statistically significant differences were apparent between all diet groups (Table A2). Compared to conventionally fed dogs, those fed vegan diets were less likely, and those fed raw meat were more likely, to be sexually intact. For dogs fed raw meat the odds of being sexually intact were more than double those of dogs fed vegan diets. Desexing differences were also significant between males and females, with males significantly more likely to be sexually intact (Table A3) [7].

#### 3.1.4 Breed size

**Fig. 4:**
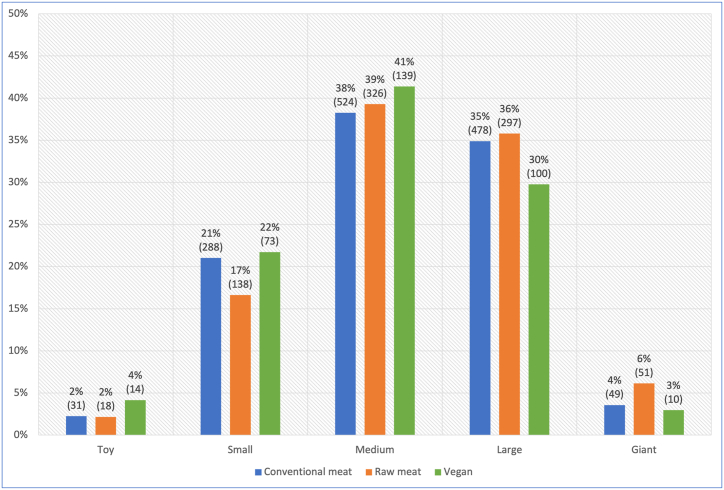
Breed sizes of 2,536 dogs fed three main diets. Note: Breed sizes were defined within the survey as: toy: ≤4.1 kg or ≤9 lb, small: 4.5–10.9 kg or 10–24 lb, medium: 11.4–22.3 kg or 25–49 lb, large: 22.7–40.5 kg or 50–89 lb, and giant: >40.9 kg or >90 lb [15].

For all 2,536 dogs, breed sizes of toy, small, medium, large or giant were reported. In all dietary groups dogs were most commonly medium-sized, followed by large or small (Fig. 4). A chi-square test of independence showed a significant association between diet type and breed size (p = 0.0031). The effect size was very small (Cramer's V = 0.068).

Considering breed sizes overall within this sample, dogs fed raw meat differed significantly from those fed conventional meat (p = 0.0116), or vegan diets (p = 0.0064). Overall, dogs fed raw meat were slightly more likely to be large to giant sized breeds. Differences between dogs fed conventional meat and vegan diets were not significant (Table A4).

#### 3.1.5 Exercise level

**Fig. 5:**
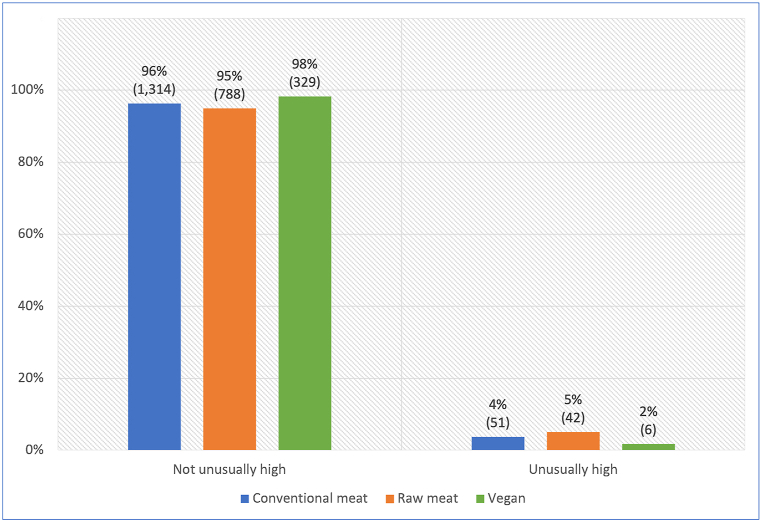
Unusually high exercise levels in 2,530 dogs fed three main diets. Note: Unusually high exercise levels were exemplified by racing greyhounds, working farm dogs, or police dogs.

Exercise levels for six dogs were not reported. Among the remaining 2,530, 3.9 % (99) were reportedly exercising at unusually high levels. Examples of such dogs included racing greyhounds, working farm dogs, or police dogs (Fig. 5). A chi-square test of independence showed a significant association between diet type and unusually high exercise level (p = 0.0297). The effect size was small (Cramer's V = 0.053). Within this sample, dogs fed vegan diets were less likely to exercise at unusually high levels, than either of the other two dietary groups. The difference between the vegan and the raw meat diet group was significant (p = 0.0174), with the former less than half as likely to exercise at unusually high levels. Differences between other dietary groups were not significant (Table A5).

### 3.2 General indicators of illness (7): effects of all control variables

The following results consider the 2,536 dogs in the three main dietary groups. The results of the regression models were interpreted based on the estimated exponentiated effects, as listed in Table 1.

**Table 1:**
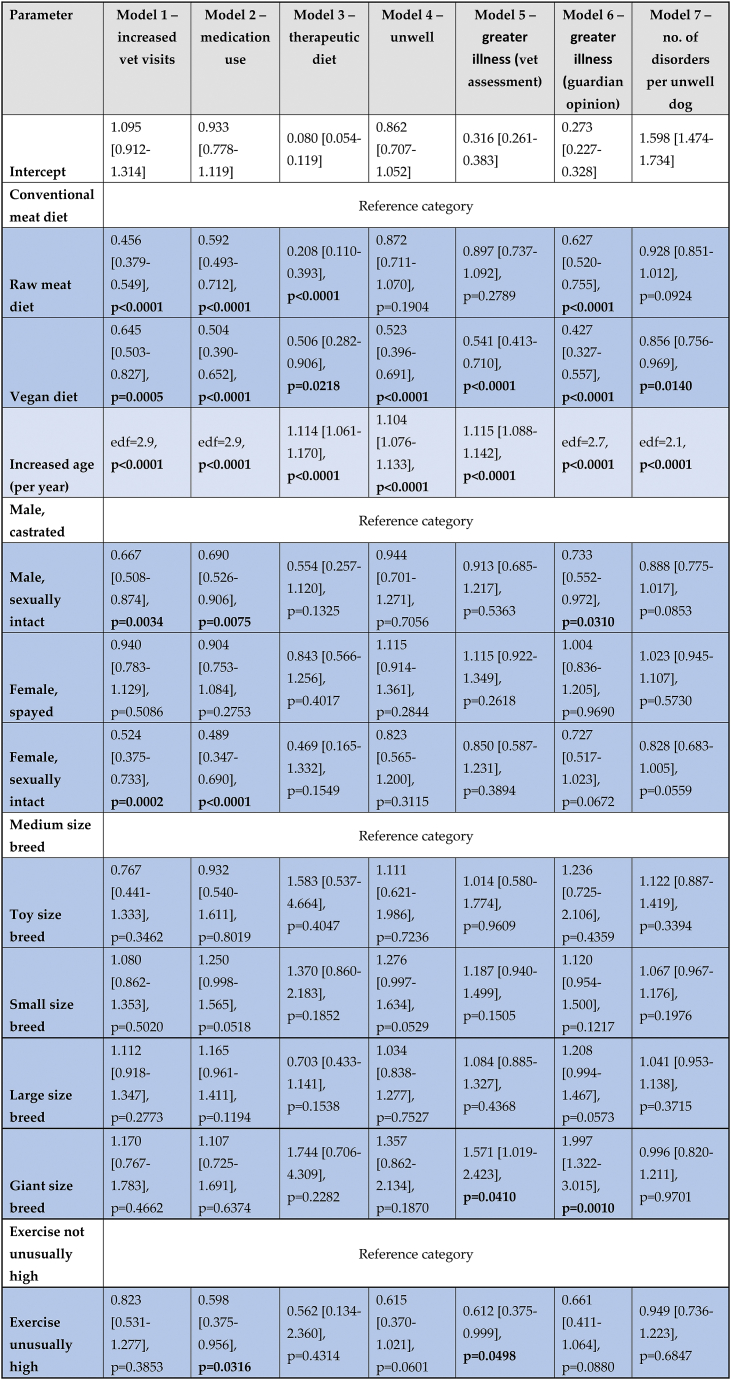
Regression model results for seven general indicators of illness among 2,536 dogs fed three main diets.

Note: Dog numbers in some groups were lower than 2,536, as described under Results. The effect of each variable is reported as ‘exponentiated effect estimate [95 % confidence interval], p-value’. Boldface indicates p < 0.05. The reference categories for variables ‘diet’, ‘sex and neuter’, ‘breed size’ and ‘exercise level’ are respectively ‘conventional meat’, ‘male, castrated’, ‘medium size’ and ‘exercise not unusually high’. The exponentiated intercept parameters encode the odds (models 1–6) of ‘unhealthy’ outcomes occurring, or the expected number of health disorders (model 7), for dogs with these reference characteristics and of average age. The effects of the other categories state average differences compared to these reference categories, or per year of age above the average (with corresponding decreases per year of decreased age). For the logistic regression models (models 1–6) these exponentiated effect estimates are odds ratios. For Quasi-Poisson model 7 the effects correspond to multiplicative changes in the expected number of health disorders per unwell dog. Some age effects were estimated nonlinearly. For these effects, the estimated degrees of freedom (edf) values are given (as a measure for how much each effect deviates from linearity) instead of a linear effect estimate. Visualisations of all control variable effect estimates and the nonlinear age effects can be found in Figs A2-A6.

**Fig. A3:**
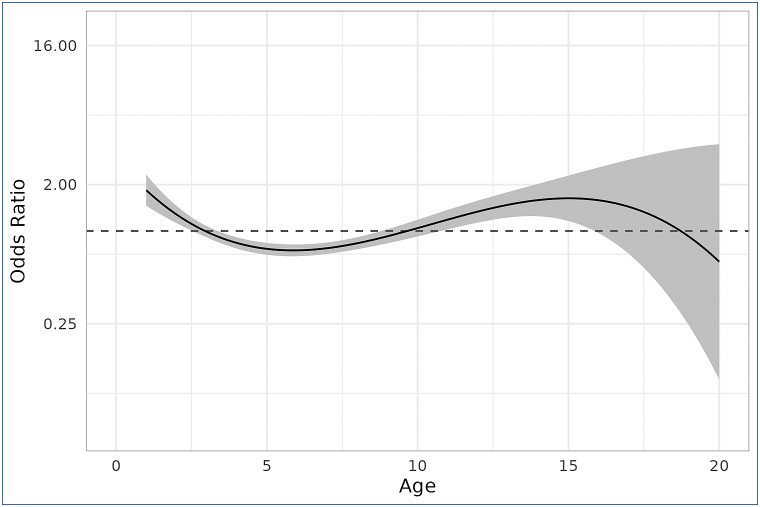
Exponentiated nonlinear age effect for the ‘increased veterinary visits’ model, on a logarithmic y-axis, in 2,520 dogs fed three main diets. Note: The solid line marks the exponentiated effect estimate, and the grey interval indicates exponentiated 95 % point-wise confidence intervals.

**Fig. A4:**
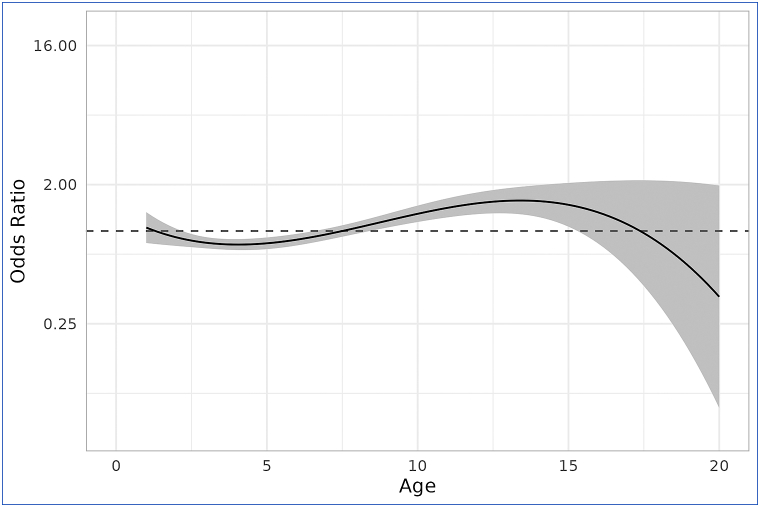
Exponentiated nonlinear age effect for the ‘medication use’ model, on a logarithmic y-axis, in 2,536 dogs fed three main diets. Note: The solid line marks the exponentiated effect estimate, and the grey interval indicates exponentiated 95 % point-wise confidence intervals.

**Fig. A5:**
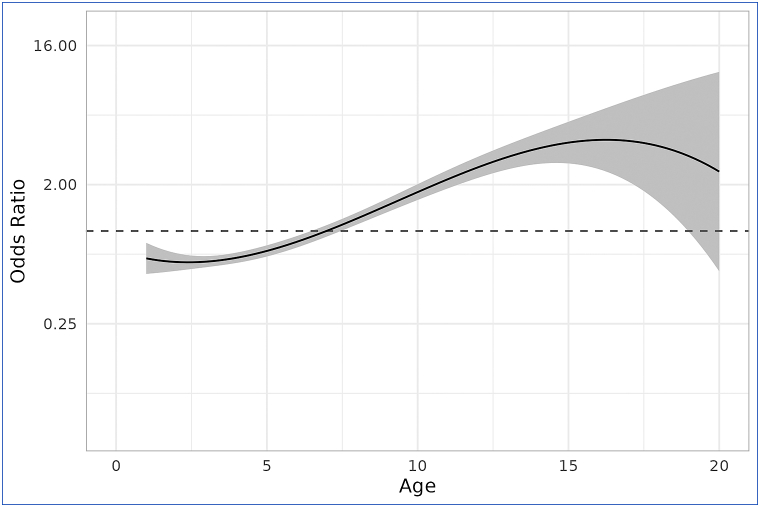
Exponentiated nonlinear age effect for the ‘greater illness (guardian opinion)’ model, on a logarithmic y-axis, in 2,530 dogs fed three main diets. Note: The solid line marks the exponentiated effect estimate, and the grey interval indicates exponentiated 95 % point-wise confidence intervals.

**Fig. A6:**
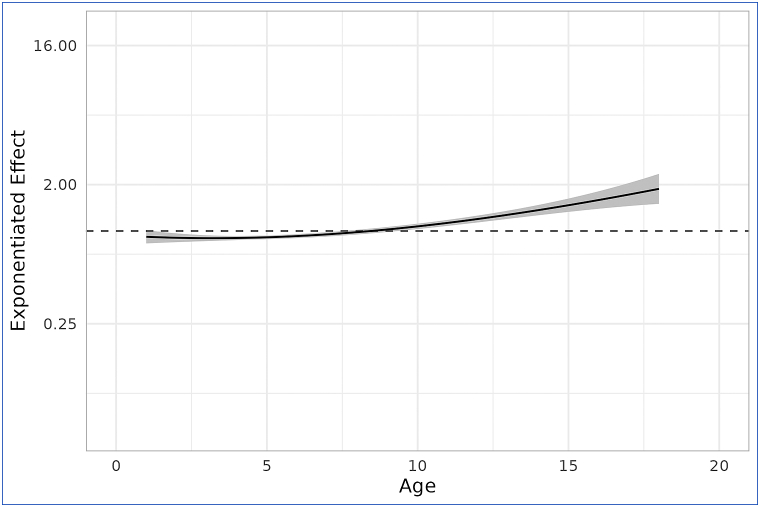
Exponentiated nonlinear age effect for the ‘number of health disorders per unwell dog’ model, on a logarithmic y-axis, in 931 dogs fed three main diets. Note: The solid line marks the exponentiated effect estimate, and the grey interval indicates exponentiated 95 % point-wise confidence intervals.

For the applicable variables, the average or most common observations respectively were: ‘meat-based diet’, ‘average age’ (∼6.2 years; for models 3, 4 and 5 (only) with linear age effects), ‘male, castrated’, ‘medium breed size’ and ‘exercise not unusually high’. These were assigned as the reference categories for these categorical variables (as indicated in Table 1). The exponentiated intercept parameters in Table 1 encode the chances (models 1–6) of the respective ‘unhealthy’ outcomes occurring, or the expected number of disorders (model 7), for dogs with these reference characteristics and of average age. Models 3–5 had linear age effects, but age effects in the remaining models 1–2 and 6–7 were non-linear, as visualised in Figures A3 – A6. The reference ages for the intercepts in these models was not ∼6.2 years, but can be determined by reading the age values where the nonlinear effect curves in Figures A3 – A6 cross the value of 1.0 (the dashed horizontal line, indicating no effect). For example, the intercept for model 1 (Fig. A3) refers to dogs aged ∼3, ∼10 or ∼18.5 years. In this case model 1 has three reference ages. Similarly, model 2 (Fig. A4) has three reference ages. However, the other models visualised each have a single reference age.

For all regression models, results can be interpreted as the multiplicative change of the outcome, independent of the exact values of all control variables (which were diet, age, sex, neutering status, breed size and exercise level). For the binary logistic models, the exponentiated effects are odds ratios (ORs), referring to a multiplicative change in the odds of the ‘unhealthy’ outcome occurring. For example, the estimated odds ratio of 0.645 for a vegan diet in the increased veterinary visits model indicates that dogs fed a vegan diet had 100 % x (1–0.645) = 35.5 % lower odds of having two or more veterinary visits in the last year (an indicator of potential illness), compared to conventional meat-fed dogs, independent of any differences in the remaining control variables between the two dietary groups. The ‘increased age’ category, indicates the increase in odds of the ‘unhealthy’ outcome occurring, per year of age above the average (models 3–5), and the estimated degrees of freedom (edf – indicating how much each effect deviates from linearity), for the remaining models. For example, for model 3, the odds of this outcome increase by a factor of 1.114, when age increases from 6.2 to 7.2 years, and by the same multiple again when age increases from 7.2 to 8.2 years. Conversely, for every year of age reduction below the average, these odds are divided by the corresponding multiple.

For the ordinal logistic models, this multiplicative change in outcome refers to a change in the odds of being in a higher category (indicating more severe illness), compared to lower categories. For the Quasi-Poisson model, the exponentiated effects refer to a multiplicative change in the expected number of health disorders per unwell dog.

Exponentiated 95 % confidence intervals are provided as measures for uncertainty. These confidence intervals are – by construction – not centered. While the confidence intervals for the raw linear effect estimates are centered, this centering is lost through the exponentiation step which enables the interpretation of the effects as odds ratios. For example, consider the effect of the vegan diet (compared to a conventional meat-based diet) in model 5 for the 'reported veterinary assessment of more severe illness'. The raw effect estimate is −0.614 [CI: −0.885 to −0.343] which is centered. Only when exponentiating does the confidence interval become non-centered. When reporting as a multiplicative change in the outcome this becomes 0.541 [CI: 0.413 to 0.710] (Table 1). When reporting as an odds reduction with the vegan diet, this becomes −45.9 % [CI: −58.7 % to −29.0 %] (Table 2). Respective odds reductions between the vegan and raw meat diet are shown in Table 3 and are calculated based on the quotient of the exponentiated effect estimates in Table 1. For example, for model 5, the odds reduction with a vegan diet (compared to a raw meat diet) is 100 % x (1 - [0.541/0.897]) = 39.7 %.

**Table 2:**
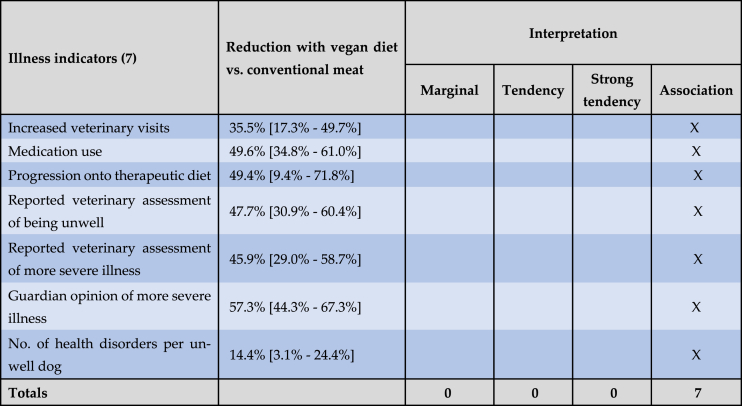
Effects on seven general indicators of illness of a vegan diet, in comparison to a conventional meat diet, among 1,706 dogs fed either diet, after controlling for canine demographic factors.

Note: Dog numbers in some groups were lower than 1,706 as described under Results. Results are reported as ‘exponentiated effect estimate [exponentiated 95 % confidence interval].’ These exponentiated effects refer to reductions in the odds of six illness indicators occurring, or to reductions in the average number of health disorders per unwell dog. Results are interpreted as “association” (a significant effect ∼10 % or stronger), “strong tendency” (a non-significant effect ∼25 % or stronger), “tendency” (a non-significant effect ∼10 % or stronger) or “marginal” (a non-significant effect <10 %) [29,30].

**Table 3:**
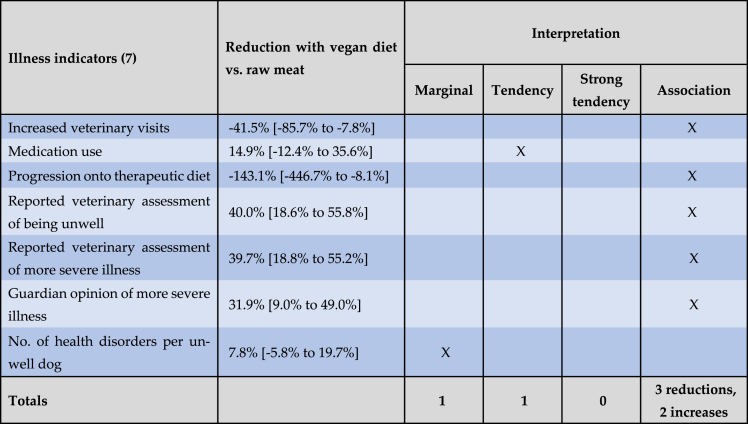
Effects on seven general indicators of illness of a vegan diet, in comparison to a raw meat diet, among 1,166 dogs fed either diet, after controlling for canine demographic factors.

Note: Dog numbers in some groups were lower than 1,166 as described under Results. Results are reported as ‘exponentiated effect estimate [exponentiated 95 % confidence interval]’, where negative percentages in a confidence interval refer to increases rather than reductions with a vegan diet. These exponentiated effects refer to reductions in the odds of six illness indicators occurring, or to reductions in the average number of health disorders per unwell dog. Results are interpreted as “association” (a significant effect ∼10 % or stronger), “strong tendency” (a non-significant effect ∼25 % or stronger), “tendency” (a non-significant effect ∼10 % or stronger) or “marginal” (a non-significant effect <10 %) [29,30].

### 3.3 General indicators of illness (7): effects of diet

**Fig. 6:**
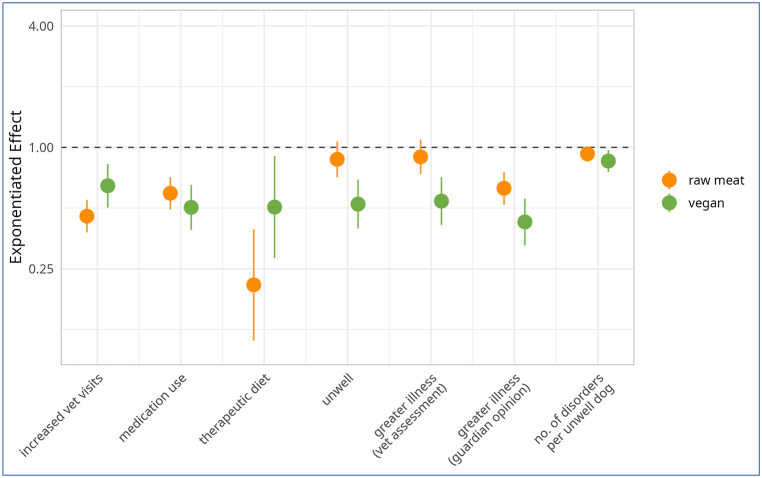
Exponentiated effect estimates for the ‘raw meat diet’ and ‘vegan diet’ coefficients, for all seven regression models, on a logarithmic y-axis, among 2,536 dogs fed three main diets. Note: Dog numbers in some groups were lower than 2,536, as described under Results. Dots mark the exponentiated effect estimates. Bars mark the corresponding 95 % confidence intervals. All effect estimates are listed in Table 2, Table 3.

The effects of the vegan diet on all seven general indicators of illness are visualised in Fig. 6 (along with the effects of the raw meat diet), and are summarised in Table 2, Table 3.

These results were then applied to an average dog with the reference characteristics described previously. For such a dog, relative reductions of occurrences of the seven general indicators of illness following a vegan diet, when compared to a conventional meat diet, are given in Table 4. For example, given the estimated results for model 2, an average dog with the reference characteristics had odds of 0.933 of receiving medication within the last year (Table 1). For an average dog fed a vegan diet, when compared to a dog fed conventional meat, the odds of receiving medication reduced by 49.6 % (Table 2), or by a factor of 0.504 (Table 1), to 0.470. Because these odds are defined as ‘Odds (‘medication use’) = P (‘medication use’)/(1 – P (‘medication use’))’, with P (‘medication use’) being the probability of receiving medication, the corresponding probability can be calculated as ‘P (‘medication use’) = 100 % x Odds (‘medication use’)/(1 + Odds (‘medication use’))’. Accordingly, for an average dog with the reference characteristics (including a conventional meat-based diet), P (‘medication use’) = 48.3 %. For a similar dog following a vegan diet, this fell to 32.0 % – a relative reduction of 33.8 %.

**Table 4:**
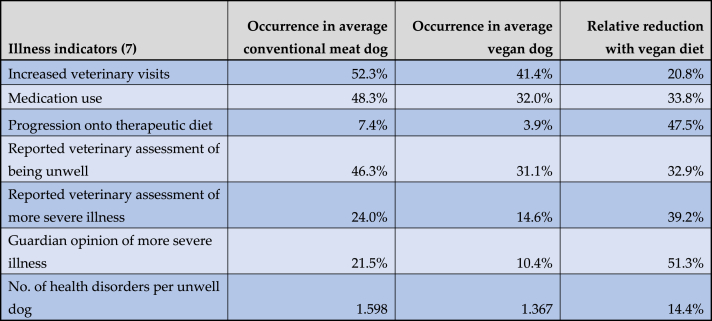
Prevalence of seven general indicators of illness among 1,706 dogs fed vegan or conventional meat-based diets, after controlling for canine demographic factors.

Note: Dog numbers in some groups were lower than 1,706, as described under Results. Average dogs were those with the reference categories defined previously. Although rounded to one decimal place, underlying results were based on data of greater precision.

Similarly, relative occurrences of these seven general indicators of illness for an average dog following a vegan diet, when compared to a raw meat diet, are given in Table 5. For example, given the estimated results for model 2, the odds of receiving medication for an average dog fed a raw meat diet, when compared to a conventional meat diet, were 0.933 × 0.592 = 0.552. For an average dog fed a vegan diet, when compared to a conventional meat diet, this was 0.933 × 0.504 = 0.470 as noted previously. The respective probabilities were 35.6 % and 32.0 %, indicating a relative reduction with a vegan diet of 10.2 %.

**Table 5:**
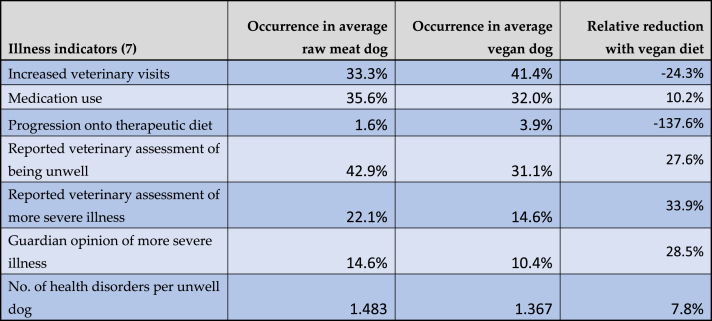
Prevalence of seven general indicators of illness among 1,166 dogs fed vegan or raw meat diets, after controlling for canine demographic factors.

Note: Dog numbers in some groups were lower than 1,166, as described under Results. Average dogs were those with the reference categories defined previously. Although rounded to one decimal place, underlying results were based on data of greater precision.

The results for the seven general indicators of illness were also studied in greater detail.

#### 3.3.1 Increased veterinary visits

**Fig. 7:**
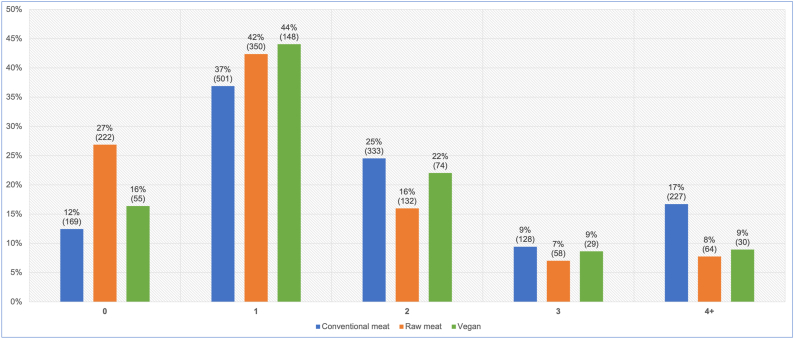
Veterinary visits of 2,520 dogs fed three main diets, in the last year. After Knight et al. [7].

Sixteen guardians were unsure of the frequency of veterinary visits within the last year. Data for the remaining 2,520 dogs are reproduced in Fig. 7. We were interested in those dogs who saw veterinarians two or more times during the previous year. This could be an indicator of ill health, as routine health checks normally occur only once annually. When comparing two groups of 0–1 visits and 2 or more visits, and controlling for dog demographic factors in the applicable regression model, differences between dietary groups were apparent. Dogs fed a vegan diet had, on average, 35.5 % lower odds – representing a 20.8 % risk reduction, of having two or more veterinary visits, compared to dogs fed a conventional meat-based diet. This effect was statistically significant (p < 0.0001). Because the odds reduction was stronger than 10 %, and statistically significant, it can be considered an *association*. Compared to dogs fed a raw meat-based diet, those fed a vegan diet also had, on average, 41.5 % greater odds – representing a 24.3 % risk increase, of having two or more veterinary visits. This effect was also statistically significant (p = 0.0124). Because the effect was stronger than 10 %, and statistically significant, it can also be considered an *association*.

#### 3.3.2 Medication use

**Fig. 8:**
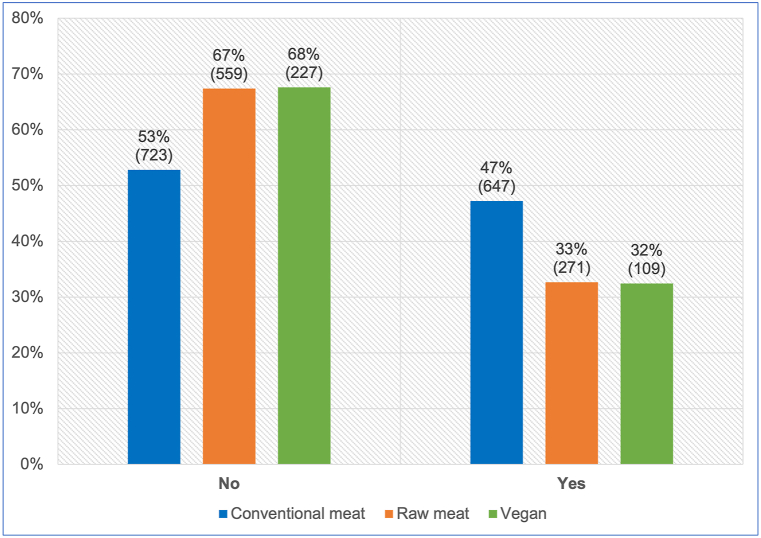
Medication use in 2,536 dogs fed three main diets. After Knight et al. [7].

All 2,536 guardians provided information about medication use in the previous year (Fig. 8). After controlling for dog demographic factors in the applicable regression model, differences between dietary groups were apparent. Dogs fed a vegan diet had, on average, 49.6 % lower odds – representing a 33.8 % risk reduction, of receiving medication (potentially indicating illness), compared to dogs fed a conventional meat-based diet. This effect was statistically significant (p < 0.0001). Because the effect was stronger than 10 %, and statistically significant, it can be considered an *association*. Dogs fed a vegan diet also had, on average, 14.9 % lower odds – representing a 10.2 % risk reduction, of receiving medication compared to dogs fed a raw meat-based diet. This effect was not statistically significant (p = 0.2554). Because the effect was stronger than 10 %, but not significant, it can be considered a *tendency*.

#### 3.3.3 Progression onto a therapeutic diet

**Fig. 9:**
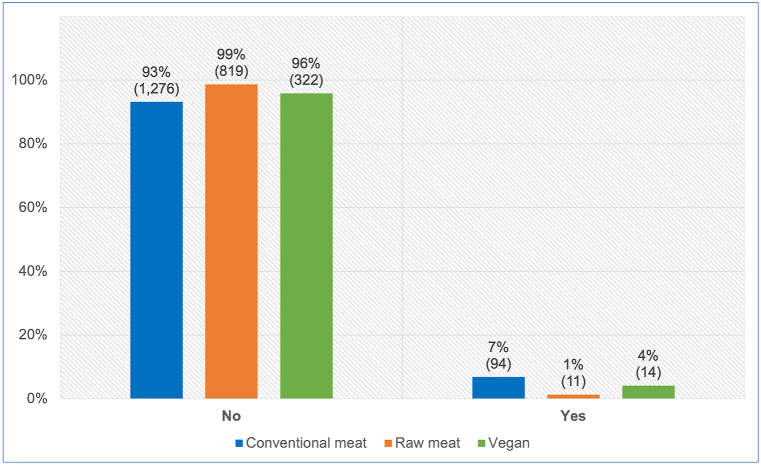
Subsequent progression onto a therapeutic diet in 2,536 dogs maintained on an initial diet as specified. After Knight et al. [7].

All 2,536 guardians provided information about whether or not their dog progressed onto a therapeutic diet, after initial maintenance on one of the three main diets (Fig. 9). After controlling for dog demographic factors in the applicable regression model, differences between dietary groups were apparent. Dogs fed a vegan diet had, on average, 49.4 % lower odds – representing a 47.5 % risk reduction, of progressing onto a therapeutic diet (potentially indicating illness), compared to dogs fed a conventional meat-based diet. This effect was statistically significant (p < 0.0001). Because the effect was stronger than 10 %, and statistically significant, it can be considered an *association*. Dogs fed a vegan diet also had, on average, 143.1 % greater odds – representing a 137.6 % risk increase, of progressing onto a therapeutic diet compared to dogs fed a raw meat-based diet. This effect was also statistically significant (p = 0.0317). Because this effect was stronger than 10 %, and statistically significant, it can also be considered an *association.*

#### 3.3.4 Reported veterinary assessment of more severe illness

**Fig. 10:**
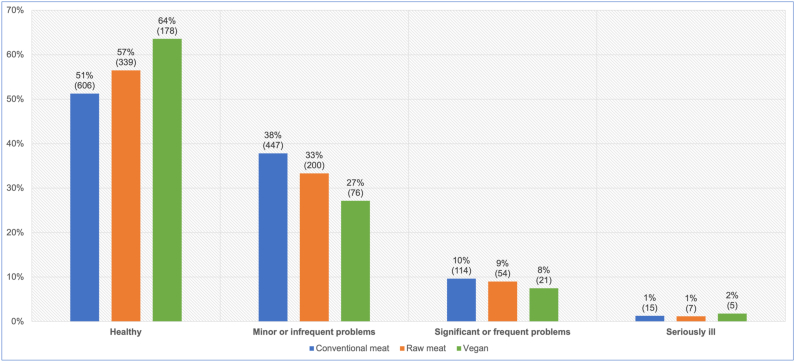
Guardian-reported veterinary assessments of the health status of 2,062 dogs fed three main diets. After Knight et al. [7].

A total of 2,074 dogs saw a veterinarian at least once in the previous year (Fig. 7). After excluding 12 ‘unsure’ respondents, the 2,062 remaining guardians were reportedly sure of the assessments of their veterinarians regarding the health status of their dogs (Fig. 10). After controlling for dog demographic factors in the applicable regression model, differences between dietary groups were apparent. Dogs fed a vegan diet had, on average, 45.9 % lower odds – representing a 39.2 % risk reduction, of reportedly being considered by veterinarians to have more severe illness, compared to dogs fed a conventional meat-based diet. This effect was statistically significant (p < 0.0001). Because the effect was stronger than 10 %, and statistically significant, it can be considered an *association*. Dogs fed a vegan diet also had, on average, 39.7 % lower odds – representing a 33.9 % risk reduction, of this outcome compared to dogs fed a raw meat-based diet. This effect was also statistically significant (p = 0.0010). Because this effect was stronger than 10 %, and statistically significant, it can also be considered an *association.*

#### 3.3.5 Guardian opinion of more severe illness

**Fig. 11:**
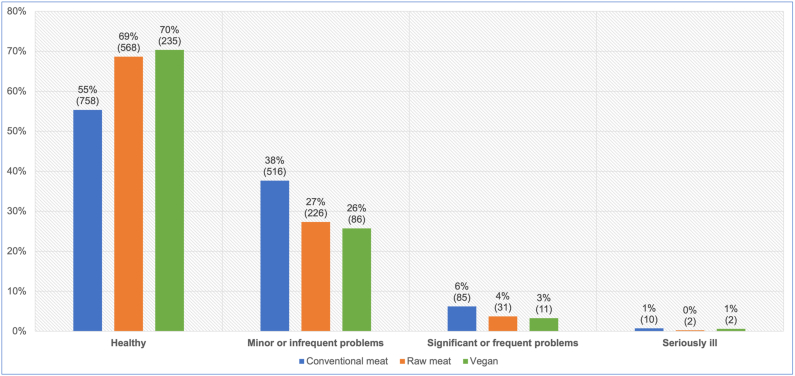
Guardian opinions of the health status of 2,530 dogs fed three main diets. After Knight et al. [7].

After excluding six ‘unsure’ responses, 2,530 guardians reported their own opinions of the health status of their dogs (Fig. 11). After controlling for dog demographic factors in the applicable regression model, differences between dietary groups were apparent. Dogs fed a vegan diet had, on average, 57.3 % lower odds – representing a 51.3 % risk reduction, of reportedly being considered by guardians to have more severe illness, compared to dogs fed a conventional meat-based diet. This effect was statistically significant (p < 0.0001). Because the effect was stronger than 10 %, and statistically significant, it can be considered an *association*. Dogs fed a vegan diet also had, on average, 31.9 % lower odds – representing a 28.5 % risk reduction, of reportedly being considered by guardians to have more severe illness compared to dogs fed a raw meat-based diet. This effect was also statistically significant (p = 0.0093). Because this effect was stronger than 10 %, and statistically significant, it can also be considered an *association.*

### 3.4 Specific health disorders (22)

As noted, 2,074 dogs saw veterinarians at least once within the preceding year (Fig. 7). In 12 cases guardians were unsure of their veterinarian's opinions. In the remaining 2,062 cases, guardians were reportedly sure of their veterinarian's opinions concerning the health of their dogs (Fig. 10). 1,123 dogs were considered to be entirely healthy. The remaining 939 dogs reportedly suffered from one or more disorders. In eight cases (conventional meat – 3, raw meat – 4, vegan – 1), no details were provided or veterinarians reportedly considered dogs to be ‘healthy’, ‘old’, or similar – i.e. not actually unwell. These cases were excluded. The remaining 931 dogs were considered further. For 161 of these dogs, details of ‘other’ disorders were reported. These were reclassified into the 18 existing, or four new disorder types, depending on details provided. In total, guardians reported that 931 dogs were considered by veterinarians to be suffering from 1,477 cases of 22 specific disorders (Table 6).

**Table 6:**
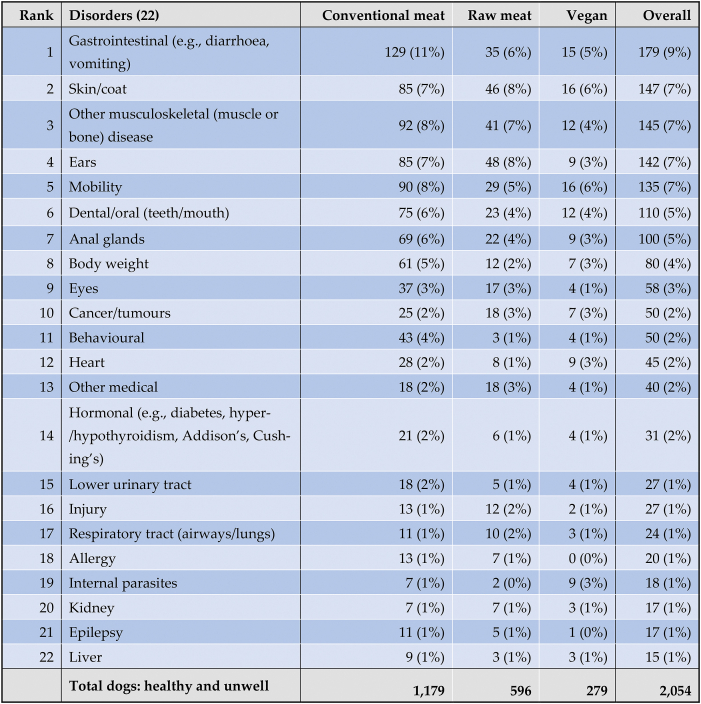
Prevalence of 22 specific health disorders or affected bodily systems in 2,054 dogs fed three main diets, based on reported opinions of veterinarians.

Note: 1,123 dogs were considered entirely healthy, with the remaining 931 suffering from one or more disorders. Relative frequencies indicate the prevalence in each diet group, and overall. Rank order reflects overall prevalence.

#### 3.4.1 Proportion of unwell dogs and average number of disorders per unwell dog

As noted, in addition to these 931 reportedly unwell dogs, the remaining 1,123 dogs were reportedly considered by veterinarians to be healthy. Forty five percent of dogs overall reportedly suffered from one or more disorders, with the average number of disorders per unwell dog being 1.59 (Table 7).

**Table 7:**
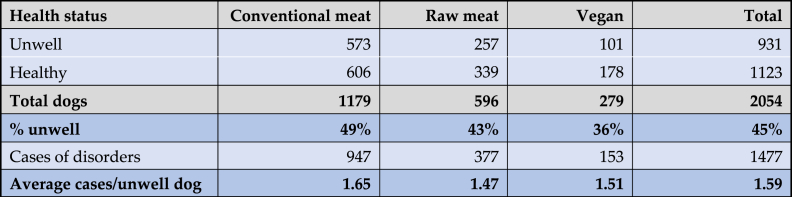
1,477 occurrences of 22 specific health disorders, in 2,054 dogs fed three main diets.

#### 3.4.2 Reported veterinary assessment of being unwell

After controlling for dog demographic factors in the applicable regression model, differences between dietary groups were apparent. Dogs fed a vegan diet had, on average, 47.7 % lower odds – representing a 32.9 % risk reduction, of reportedly being considered unwell by veterinarians, compared to dogs fed a conventional meat-based diet. This effect was statistically significant (p < 0.0001). Because the effect was stronger than 10 %, and statistically significant, it can be considered an *association*. Dogs fed a vegan diet also had, on average, 40.0 % lower odds – representing a 27.6 % risk reduction, of being considered unwell compared to dogs fed a raw meat-based diet. This effect was also statistically significant (p = 0.0010). Because this effect was stronger than 10 %, and statistically significant, it can also be considered an *association.*

#### 3.4.3 Number of disorders per unwell dog

The number of disorders per unwell dog is shown in Table 8. For the 931 dogs reportedly assessed by veterinarians to be suffering from a disorder, dogs fed raw meat and vegan diets both had less disorders, on average, than those fed a conventional meat-based diet. The mean number of disorders were: conventional meat-based = 1.65 (sd 0.991), range 1–8, raw meat-based = 1.47 (sd 0.968), range 1–7, and vegan diets = 1.51 (sd 0.795), range 1–5 (Table 7, Table 8).

**Table 8:**
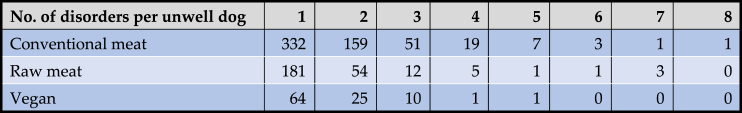
Number of disorders per unwell dog, among 931 unwell dogs fed three main diets.

After controlling for dog demographic factors in the applicable regression model, differences between dietary groups were apparent. Dogs fed a vegan diet had, on average, 14.4 % fewer health disorder cases, compared to dogs fed a conventional meat-based diet. This effect was statistically significant (p = 0.0140). Because the effect was stronger than 10 %, and statistically significant, it can be considered an *association*. Dogs fed a vegan diet also had, on average, 7.8 % fewer health disorder cases, compared to dogs fed a raw meat-based diet. This effect was not statistically significant (p = 0.2476). Because this effect was less than 10 %, and not significant, it can be considered *marginal*.

#### 3.4.4 Prevalence of 22 specific health disorders

**Fig. 12:**
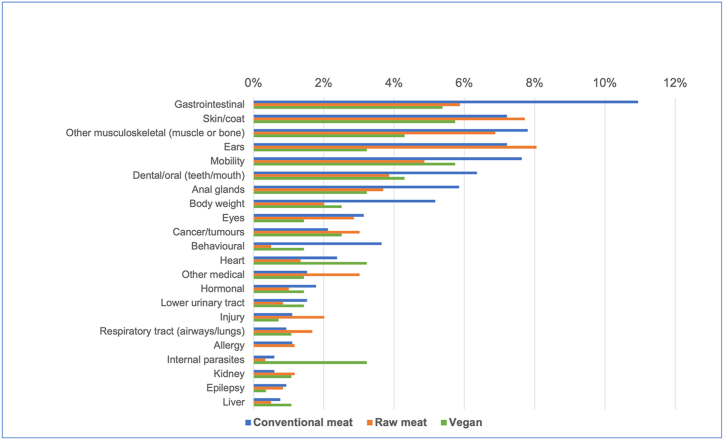
Prevalence of 22 specific disorders or affected bodily systems in 2,054 dogs fed three main diets, based on reported assessments of veterinarians [7]. Note: Vertical axis order reflects overall prevalence of disorders (combining all diets).

The prevalence of these 22 specific disorders in these 2,054 dogs is indicated in Table 9 and Fig. 12.

**Table 9:**
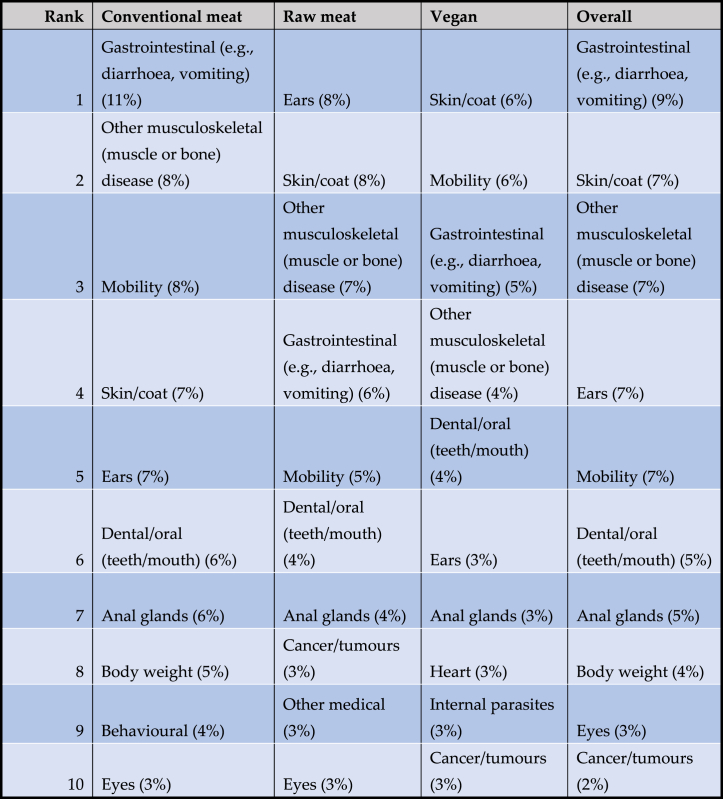
The 10 most common disorders or affected bodily systems among 2,054 dogs fed three main diets, and overall, based on reported assessments of veterinarians [7].

Note: Percentages provide the prevalence of each disorder within each dietary group, and overall.

#### 3.4.5 Differences between dietary groups

Based on probability of occurrence, the 10 most common health disorders found within each dietary group are listed in Table 9. After controlling for canine demographic factors in each of the 22 applicable regression models, some significant differences between dietary groups were detected, in the prevalence of certain disorders. There are indicated in Table 10 and summarised in Table 11.

**Table 10:**
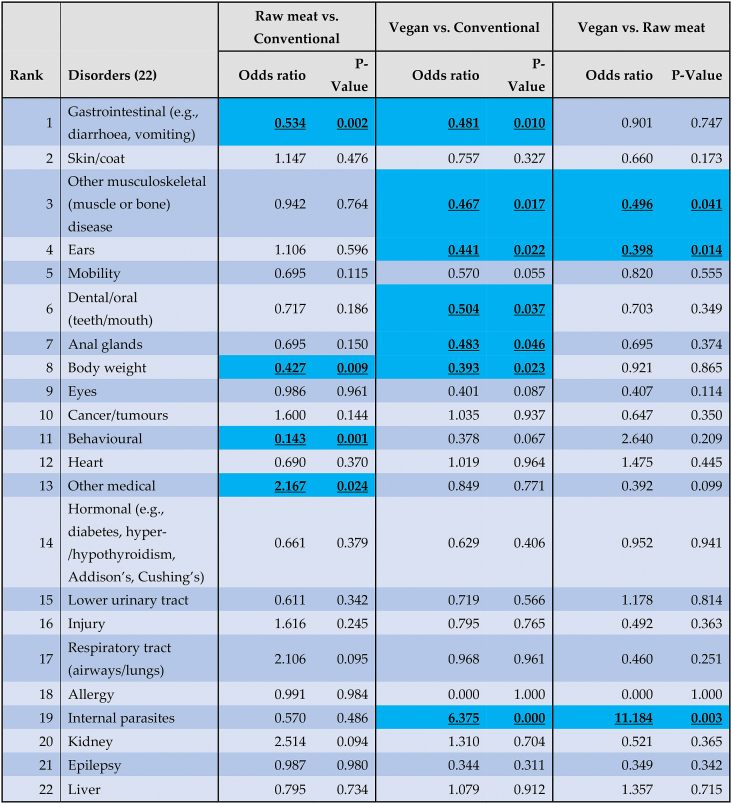
Differences in the odds of 22 specific disorders or bodily system effects occurring among 2,054 dogs fed three main diets, based on reported assessments of veterinarians, after controlling for canine demographic factors.

Note: Ranking is based on overall prevalence of disorders (combining all diets). Statistically significant differences between diet groups are highlighted.

**Table 11:**
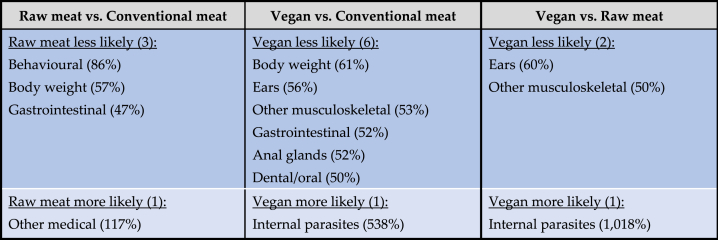
Health disorders or bodily system effects with significantly different odds of occurrence among 2,054 dogs fed three main diets, based on reported assessments of veterinarians, after controlling for canine demographic factors.

Note: Percentage alterations in odds of health disorder occurrence are provided. E.g., dogs fed vegan diets had 61 % lower odds of suffering from body weight disorders, compared to dogs fed conventional meat-based diets.

For five disorders, guardians had the option to provide additional information. For skin/coat problems, further information was provided in 140 of 147 cases. The most common causes were (in order), atopic/allergic dermatitis (inflamed skin due to allergies), moist dermatitis, and pruritis (itchiness) of unspecified origin. For mobility problems, further information was provided in 123 of 135 cases. The most common causes were (in order) osteoarthritis/arthritis, and a variety of 'other' causes. For dental/oral problems, further information was provided in 109 of 110 cases. The most common causes were (in order), dental calculus/plaque/tartar, gingivitis, and a variety of ‘other’ causes, particularly damaged, broken or worn teeth. For body weight problems, all respondents (80) identified whether dogs were over- or underweight. Eighty five percent (68) of dogs were reportedly overweight, and 15 % (12) reportedly underweight. For eye problems, further information was provided in 57 of 58 cases. Among the most common causes were eye ulcers and related conditions such as dry eye and entropion, conjunctivitis, infections, blindness/vision loss and cataracts.

## 4 Discussion

### 4.1 Canine demographic factors relevant to health

We examined differences between diet groups in canine demographic factors that may affect health outcomes. These included age, sex, neutering status, breed size and unusually high exercise levels.

With respect to age, the age distributions within each dietary group appeared to broadly resemble those of normal dogs [20,32]. However, dogs fed vegan diets were, on average, 1.8 years older than dogs fed raw meat, and 1.0 years older than dogs fed conventional meat-based diets. These differences were statistically significant. This was exemplified by considering the first five years of life (Fig. 2). Dogs fed raw meat were most likely, and those fed vegan diets, least likely, to be aged <5. Younger dogs generally have better health. Hence, this difference can be expected to have positively influenced the health outcomes of dogs fed raw meat diets, and negatively influenced those of dogs fed vegan diets, within our sample. The impacts of average age differences are more clearly contextualised by considering that one year of canine life equates to many years of human life [33].

With respect to sex/neuter status, males comprised just over half of the 2,536 dogs within our sample (Fig. 3). A 2016 study of 22,333 UK dogs [20], similarly found just over half to be male. Although variations in sex distributions within our sample were not significant, statistically significant differences between dietary groups did exist with respect to desexing. Within our sample, around three quarters of all dogs were desexed (Fig. 3). In contrast, O'Neill et al. [20], found 45 % of their dogs were desexed. Within our sample, male dogs were less likely to be desexed (Table A3). O'Neill et al. [20], similarly found desexing to be slightly less common among males. Our study also found desexing to be least common for dogs fed raw meat diets, and most common for dogs fed vegan diets. These differences were statistically significant between all dietary groups (Table A2). This could be because guardians feeding raw meat diets were less likely to visit veterinarians (Fig. 7). It follows that such people are less likely to receive or comply with veterinary advice, including routine preventative healthcare recommendations such as desexing.

With respect to breed, we anticipated that small numbers within certain breed groups would limit our ability to statistically analyse subsequent results, and so elected to consider breed size groups (which combine multiple breeds of similar sizes). Breed sizes within our sample appeared representative of the domestic dog population, with the most common size in all diet groups being ‘medium’, and the least common sizes being the ‘toy’ and ‘giant’ breed groups at the extreme ends of the size range (Fig. 4). Overall, dogs fed raw meat were slightly more likely to be medium to giant sized breeds, which could affect health outcomes related to body mass, such as some musculoskeletal conditions. Other differences between dietary groups were not statistically significant.

Exercise levels vary widely amongst domesticated dogs, and we generally did not attempt to differentiate between these. We did, however, attempt to identify dogs exercising at unusually high levels, such as racing greyhounds, working farm dogs, or police dogs, whose exercise levels might differ from those of dogs within normal households, in medically significant ways (e.g., increased risks of injury and lameness, but also increased cardiovascular fitness). Within our sample 4 % of dogs were exercising at unusually high levels. This was most common for dogs fed raw meat (Fig. 5). These dogs were more than twice as likely as dogs fed vegan diets to be exercising at unusually high levels. Other differences between dietary groups were not statistically significant.

In short, there were multiple significant differences between diet groups with respect to canine demographic factors that could potentially alter health outcomes. Accordingly, our regression models controlled for possible effects of age, sex and neutering status, breed size and unusually high exercise levels, on the seven general indicators of illness, and the 22 specific health disorders, that we studied.

### 4.2 General indicators of illness (7)

#### 4.2.1 Increased veterinary visits

As noted previously [7], routine health checks and administration of vaccinations and parasiticides as appropriate, are normally conducted annually, at least in the UK [[34], [35], [36]]. Seventy-one per cent of our respondents were UK-based. Veterinary visits may increase for puppies or geriatric animals, but these comprised a low proportion of our sample (Fig. 2). Hence, one or less veterinary visits in the previous year would normally be consistent with good health, whereas two or more visits could indicate a health problem. After controlling for canine demographic factors via the applicable regression model, it was apparent that average dogs fed vegan diets had 36 % lower odds – representing a risk reduction of 21 %, of having two or more veterinary visits, than dogs fed conventional meat-based diets (Table 2, Table 4). Interestingly however, average dogs fed vegan diets also had 42 % greater odds – representing a risk increase of 24 %, of having two or more veterinary visits, than dogs fed raw meat (Table 3, Table 5). In both cases these differences were statistically significant.

The apparent superiority of raw meat diets when considering increased veterinary visits, appears to be largely due to a major increase in the proportion of dogs who did not see a veterinarian at all in the last year, compared to the other two dietary groups. Twenty-seven per cent of dogs fed raw meat fell within this group, compared to 12 % fed conventional and 16 % fed vegan diets respectively (Fig. 7). This could indicate a lack of healthcare problems in dogs fed raw meat. However, most veterinarians are opposed to the feeding of raw meat diets, due to repeated studies showing such diets have substantial risks of pathogen contamination and nutritional deficiencies [[37], [38], [39], [40], [41], [42], [43], [44], [45], [46]]. The opposition of most veterinarians to dietary choices these guardians make for their dogs, may make these guardians less likely to visit veterinarians, and to accept veterinary advice. Guardians feeding raw meat diets appear more inclined to gather information from other sources, including online resources of variable quality [47]. Hence, in this case of raw meat diets, veterinary visits are probably being lowered for reasons unrelated to canine health.

#### 4.2.2 Medication use

Medication use was also considered an indicator of a possible health problem. Medication use was significantly more common among dogs fed conventional meat diets (Fig. 8). After controlling for canine demographic factors via the applicable regression model, it was apparent that average dogs fed vegan diets had 50 % lower odds – representing a risk reduction of 34 %, of receiving medication, than those fed conventional meat. This effect was statistically significant (Table 2, Table 4). Average dogs fed vegan diets also had 15 % lower odds – representing a risk reduction of 10 %, of receiving medication, than those fed raw meat. This effect was not statistically significant (Table 3).

As with the ‘increased veterinary visits’ indicator of illness, medication use may also be affected by non-medical factors. Veterinary clinics are primary sources of medications for companion animals, and are near-exclusive sources of prescription medicines. In most jurisdictions animals receiving prescription medications must be examined at least once within the preceding year by a veterinarian. Hence, the markedly decreased proportion of veterinary visits by dogs fed raw meat (Fig. 7) may also have decreased the proportion medication use among these dogs.

#### 4.2.3 Progression onto a therapeutic diet

Similarly to medication use, progression on to a therapeutic diet may also indicate a medical problem. Such progression after initial maintenance on a conventional meat, raw meat or vegan diet for at least one year, was reported by 5 % (119/2,536) of respondents overall (Fig. 9). After controlling for canine demographic factors via the applicable regression model, it was apparent that average dogs fed vegan diets had 49 % lower odds – representing a risk reduction of 48 %, of progressing onto a therapeutic diet, compared to dogs fed conventional meat. This effect was statistically significant (Table 2, Table 4). However, average dogs fed vegan diets had 143 % greater odds – representing a risk increase of 138 %, of such progression onto a therapeutic diet, compared to dogs fed raw meat. This effect was also statistically significant (Table 3, Table 5).

Therapeutic diet use was dramatically lower in dogs fed raw meat, compared to other dietary groups. Similarly to medications, veterinary clinics are also the main sources of therapeutic diets, and the unusually low rates of visits to veterinary clinics for dogs fed raw meat (Fig. 7) likely also lowered the proportion of these dogs who received such diets. Additionally, by 2024 few therapeutic diets were marketed as ‘vegan’, and to the authors' knowledge, none as ‘raw meat’ diets. These factors likely explain the dramatically lowered rates of therapeutic diet use, especially among dogs initially fed raw meat.

#### 4.2.4 Reported veterinary assessment of being unwell

Based on reported veterinary assessments, 45 % of 2,054 dogs were considered unwell (Table 7). In contrast, O'Neill et al. [20] reported that 66 % of 22,333 UK dogs suffered from at least one health disorder during 2016. Lower illness rates in our study may be due to the higher proportion of dogs fed vegan or raw meat diets, following our active efforts to recruit guardians who fed such diets. Our results indicated that illness rates were lower among these groups: 43 % among dogs fed raw meat, and 36 % amongst those fed vegan diets (Table 7). After controlling for canine demographic factors via the applicable regression model, it was apparent that average dogs fed vegan diets had 48 % lower odds – representing a risk reduction of 33 %, of being considered unwell, than dogs fed conventional meat (Table 2, Table 4). This difference was statistically significant. Average dogs fed vegan diets also had 40 % lower odds – representing a risk reduction of 28 %, of being considered unwell, compared to those fed raw meat (Table 3, Table 5). This difference was also statistically significant.

#### 4.2.5 Reported veterinary assessment of more severe illness

After controlling for canine demographic factors via the applicable regression model, average dogs fed vegan diets had 46 % lower odds – representing a risk reduction of 39 %, of reportedly being considered by veterinarians to be more severely ill, than dogs fed conventional meat (Table 2, Table 4). This difference was statistically significant. Average dogs fed vegan diets also had 40 % lower odds – representing a risk reduction of 34 %, of being considered by veterinarians to be more severely ill, compared to dogs fed raw meat (Table 3, Table 5). This difference was also statistically significant.

#### 4.2.6 Guardian opinion of more severe illness

After controlling for canine demographic factors via the applicable regression model, average dogs fed vegan diets had 57 % lower odds – representing a risk reduction of 51 %, of being considered by guardians to be suffering from more severe illness, than dogs fed conventional meat (Table 2, Table 4). This difference was statistically significant. Average dogs fed vegan diets also had 32 % lower odds – representing a risk reduction of 29 %, of being considered by guardians to be suffering from more severe illness, compared to dogs fed raw meat (Table 3, Table 5). This difference was also statistically significant.

#### 4.2.7 Consistency of guardian opinions with reported veterinary assessments

Assessments of illness severity were similar between guardians and reported veterinary assessments, albeit with a shift of ∼5–10 % in most groups, toward guardians considering dogs to be healthier (Fig. 10, Fig. 11). 74.9 % of guardians agreed with reported veterinary assessments, but 15.2 % felt their dog was healthier, and 9.9 % felt their dog was less healthy, than reported veterinary assessments. Hence, although most guardians were in agreement, those who disagreed with their veterinarians were more likely to view their dogs as healthier. This result validated our decision to use reported veterinary assessments, rather than guardian opinions, to provide more conservative assessments of wellness, when considering the prevalence of the 22 most common health disorders, and general health indicators arising from these – notably veterinary assessment of being unwell, and the number of health disorders per unwell dog.

#### 4.2.8 Number of health disorders per unwell dog

Unwell dogs suffered from up to eight health disorders (Table 8), although the average number per unwell dog was 1.59 (Table 7). After controlling for canine demographic factors via the applicable regression model, unwell dogs fed vegan diets had 14 % fewer health disorder cases than dogs fed conventional meat. This was statistically significant (Table 2). Unwell dogs fed vegan diets also had 8 % fewer health disorder cases than dogs fed raw meat. This effect was not statistically significant (Table 3).

#### 4.2.9 General illness indicators overall

**Fig. 13:**
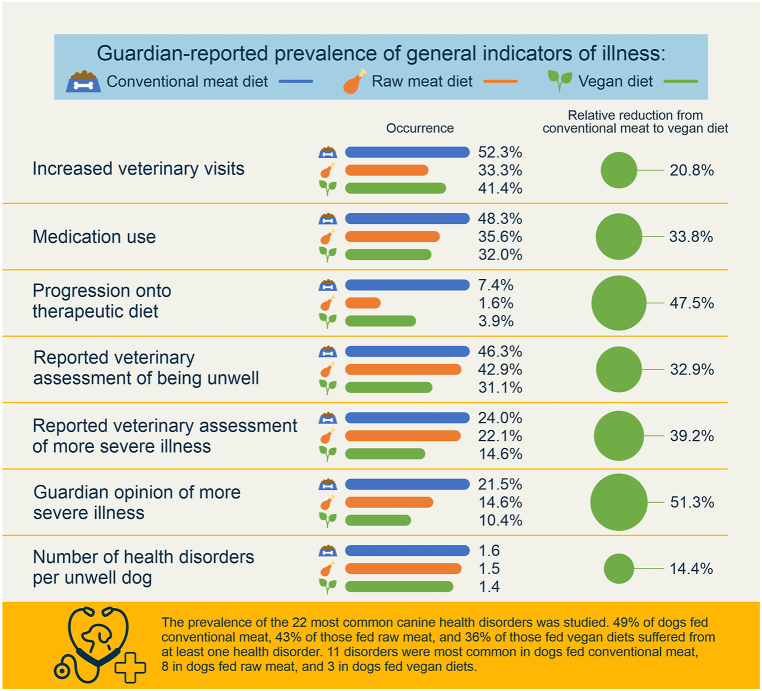
Summary of the health effects of vegan and meat-based diets for dogs with average characteristics.

After controlling for canine demographic factors via the applicable regression models, dogs fed vegan diets had superior health outcomes to those fed conventional meat, for all seven general illness indicators studied. Fig. 13 summarises the effects of the vegan diets for dogs with average characteristics. In six of seven cases lowered odds of illness indicators were substantial (36 %–57 %), and for all illness indicators, these differences were statistically significant (Table 2). Dogs fed vegan diets also had superior health outcomes to those fed raw meat, for five of seven general illness indicators studied. In three of these five cases lowered odds of illness indicators were both substantial (32 %–40 %) and statistically significant (Table 3). Collectively, dogs fed vegan diets had better health outcomes than either of the meat-based diets (Table 12), and this trend was consistent.

**Table 12:**
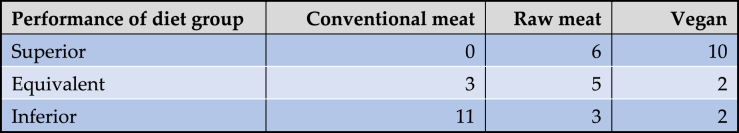
Performance of each dietary group compared to the other two diets, with respect to seven general indicators of illness, after controlling for canine demographic factors.

Note: Comparing each diet group with the other two diets, for seven general indicators of illness, results in 14 comparisons; hence each column totals 14. Performance was considered superior/inferior if differences were associations or strong tendencies. Performance was considered equivalent if differences were tendencies or marginal tendencies. As discussed under Methodology, results were interpreted as “association” (when a significant effect ∼10 % or stronger), “strong tendency” (when a non-significant effect ∼25 % or stronger), “tendency” (when a non-significant effect ∼10 % or stronger) or “marginal” (when a non-significant effect <10 %) [29,30].

This overall result was significantly different from apparent results prior to controlling for canine demographic factors (Table 13). This was anticipated. As noted, there was a statistically significant, medium-sized difference between the average ages of dogs fed raw meat and vegan diets, with the former on average 1.8 years younger than the latter. As noted, each year of dog life is equivalent to many years of human life [33], from a veterinary healthcare perspective. This is likely to have improved the general indicators of illness among dogs fed raw diets, and to have lowered the apparent prevalence of certain specific disorders [20]. We noted in our earlier study (prior to controlling for demographic factors) (Table 14), that certain specific disorders appeared less prevalent in dogs fed raw meat – five disorders, when compared to dogs fed conventional diets, and one disorder, when compared to dogs fed vegan diets. However, the younger ages of dogs fed raw meat, on average, are likely to have lowered the prevalence of at least three of these six disorders (dental/oral, body weight and mobility disorders) [48]. After controlling for effects of age and other demographic factors via our regression models, in two cases (dental/oral and mobility disorders) the apparent advantage of the raw meat diet disappeared altogether. In the case of body weight it remained, but the effect size reduced.

**Table 13:**
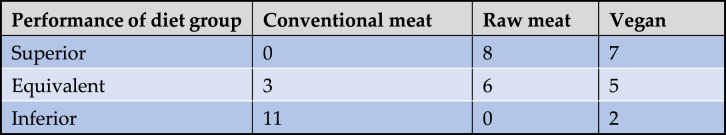
Performance of each dietary group compared to the other two diets, with respect to seven general indicators of illness, prior to controlling for canine demographic factors ([7], Table 20).

Note: Comparing each diet group with the other two diets, for seven general indicators of illness, results in 14 comparisons; hence each column totals 14.

**Table 14:**
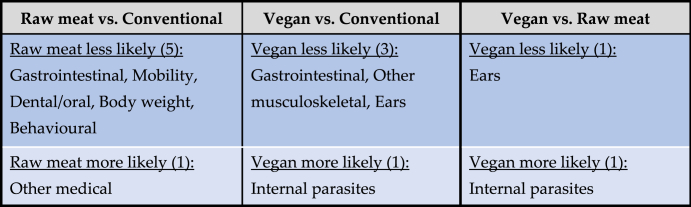
Health disorders or bodily system effects with significantly different prevalences between dietary groups, among 2,054 dogs, based on reported assessments of veterinarians, prior to controlling for canine demographic factors (after [7], Table 19).

Note: Within each cell, health disorders or affected bodily system effects are not listed in any particular order.

It is also important to recall that guardians of dogs fed raw meat are less likely to visit veterinarians (Fig. 7), and to receive or comply with veterinary advice or treatments (exemplified by lowered rates of neutering – Fig. 3). This is very probably because they disagree with veterinarians on a fundamental matter – most veterinarians oppose their dietary choices to feed raw meat. This effect is likely to lower some apparent illness indicators (e.g., increased veterinary visits, medication and therapeutic diet use), for reasons unrelated to health. Additionally, to the authors’ knowledge there were no raw meat therapeutic diets available by 2024. This would also have reduced the likelihood that dogs initially fed raw meat would progress onto a therapeutic diet when medically warranted, where dog guardians were committed to feeding raw meat. Collectively, these factors are likely to have lowered multiple apparent indicators of illness among dogs fed raw meat, for reasons unrelated to health. Hence, the true health status of dogs fed raw meat was likely to have been significantly worse than suggested by the illness indicators we studied.

#### 4.2.10 Consistency with prior related studies: general health

Our results are largely consistent with those of other studies in this field. Since we conducted our 2020 survey, key new studies have been published, supporting the feeding of nutritionally-sound vegan dog food.

Dodd et al. [49] studied 31 dogs fed an experimental vegan diet (‘PLANT’) and 30 fed a commercial meat-based diet (‘MEAT’) for 3 months. Vitamin D levels, bone composition and other health parameters were studied. They concluded that, “Bone mineral content and density did not differ from baseline values. Health status was maintained in dogs fed PLANT and vitamin D2 [which is sourced from non-animal sources] appeared efficacious in maintaining serum total vitamin D concentrations and bone mineralisation. Findings support the hypothesis that PLANT was comparable to MEAT for maintenance of healthy adult dogs for at least 3 months …”.

Linde et al. [50] studied 15 dogs fed vegan diets (V-Dog ‘Kind Kibble’) for one year after previously being fed meat-based diets. They evaluated clinical, haematological (blood cells etc.), and nutritional parameters at 0, 6, and 12 months, including complete blood count (CBC), blood chemistry, cardiac biomarkers, plasma amino acids, and serum vitamin concentrations. All dogs maintained their health status. Three who had been overweight or obese lost weight. Blood results confirmed the diet provided all essential amino acids, and for several nutrients blood levels increased. In some cases previous deficiencies reversed, without supplement use.

Dodd et al. [51] surveyed 1,189 dog guardians, including 357 feeding solely vegan diets who had used these diets for an average of three years. After similarly using regression analyses to control for medically-relevant canine demographic factors, Dodd et al. found that vegan dogs were reportedly more likely to be in very good health. They were less likely to experience several specific disorders (see ‘Specific health disorders’ following). No health disorders became more likely, and the reported longevity of previously-owned dogs increased by 1.5 years when fed purely vegan diets.

Davies [53] surveyed 100 dog guardians who had changed their dogs' diets to a nutritionally complete vegan dog food designed by veterinarians in the UK. Clear improvements after 3–12 months were reported in a range of disorders (see ‘Specific health disorders’ following).

Cavanaugh et al. [53] performed physical examinations, and conducted blood and urine tests and cardiac ultrasounds, on 34 previously meat-based dogs who were fed vegan dog food (V-dog) for three months, along with four control dogs. The dogs transitioned onto the vegan diets did not develop any deficiencies in taurine or essential amino acids. Instead, their taurine concentrations increased, and were higher than the whole blood taurine concentrations of the control dogs who were fed meat-based dog food. Results from clinicopathologic (haematology – blood cell type analysis, biochemistry – blood enzyme analysis, and urinalysis) and echocardiographic (cardiac ultrasound) tests also indicated the health of the dogs was maintained after 12 weeks fed the vegan diet.

Kiemer [54] surveyed 250 dog guardians, of whom 84.8 % used vegan dog food, 14.0 % used variations of vegetarian diets, and 1.2 % used meat-based commercial dog food. Blood nutrient levels were analysed in 40 dogs. Of these, 20 were fed a meat-based diet and 20 were fed a vegan diet for 2.15 years on average. Nutrients tested included total protein, calcium, magnesium, iron, taurine, l-carnitine, vitamin B12 and folic acid, although taurine and l-carnitine were only tested in three dogs due to higher costs. Only two dogs in the vegan group experienced nutritional deficiencies (decreased folic acid). These belonged to the same owner, and were reportedly diagnosed with giardia infection shortly after blood sampling. Giardiasis can interfere with intestinal uptake of nutrients [48] (p. 549), such as folic acid. In comparison, 11 dogs in the meat-based group had deficiencies.

Eight dogs were also placed on a six-week feeding trial, with one group (n = 4) fed a vegan diet, and the control group (n = 4) fed a meat-based diet. Blood tests were performed prior to and at the end of the trial, for the same nutrients, excluding calcium and magnesium, which were not tested due to costs. Within the vegan trial group most nutrient values did not significantly change, but several deficiencies resolved.

When jointly considering the 20 dogs maintained on vegan diets long-term, and the four dogs maintained on vegan diets during the six-week feeding trial (n = 24), 66.7 % of dog guardians reported that switching to a vegan diet resulted in no change in health status. Health improvements were reported by 29.2 %, health decreases by none, and 4.2 % were unsure.

Among the 250 dog guardians surveyed, dog health disorders were reportedly absent in 76.8 % and present in 23.2 %. Ongoing medication was not used by 87.2 %, was used by 11.2 %, and was not reported by 1.6 %. A variety of health changes while feeding a plant-based diet were reported by 54 %, with the great majority reporting improvements. Smaller guardian groups provided information about various conditions (see ‘Consistency with prior related studies: specific health disorders’).

Kiemer concluded that, “Blood chemistry analysis and physical examinations of the vegan dogs in this study together clearly indicate that a vegan diet can be healthy and adequate for dogs, and in some cases, even improve overall health. The additional data collected from 250 dog owners feeding a plant-based diet strongly supported this conclusion.”

Semp [55] also demonstrated equivalent or superior health outcomes for vegan dogs after studying vegan companion animals in Austria, Germany and Switzerland. A questionnaire was completed by 174 dog and 59 cat guardians, some of whom had both species. Participating dogs had eaten vegan diets for six months to seven years, with a mean of 2.83 years. Clinical examinations and blood tests were conducted on 20 randomly selected dogs. No diet-related clinical abnormalities were detected. Haematological (complete blood count) and biochemical (liver, kidney, and pancreatic) parameters were assessed, as well as levels of magnesium, calcium, iron, total protein, folic acid, vitamin B12, and carnitine. The serum total protein of all dogs was within normal ranges. No significant differences were evident in any tested parameters, compared to dogs fed a conventional diet. Not even the dogs fed a homemade supplemented diet (2 of 20) had any significant deviations. Some of those guardians in these two studies also reported a range of specific health benefits associated with vegan diets, as noted in the following (‘Specific disorders’).

Brown et al. [56] (n = 12) studied clinical examination and haematology data from dogs undergoing sustained, high exercise levels (sprint-racing Siberian huskies). These dogs were fed either a commercial meat-based diet recommended for active dogs (n = 6), or a vegetarian diet formulated to the same nutrient specifications (n = 6). The dogs were fed these diets for 16 weeks, including 10 weeks of competitive racing. Blood tests were conducted on four occasions, and veterinary health checks on three occasions. All dogs were assessed as being in excellent physical condition, and none developed anaemia or other detectable health problems.

In 1994, People for the Ethical Treatment of Animals (PETA) conducted a systematic survey of the health of 300 North American dogs fed vegan (65 % - 196/300), or vegetarian (35 % - 104/300) diets. Dogs ranged in age from puppies to 19 years, and a wide range of breeds were represented. Eighty-two per cent of dogs fed vegan diets for at least five years were considered to be in good to excellent health. Amongst those fed vegetarian diets for at least five years, this proportion was 77 % [57].

Only one study appears to have been published to date that does not support the feeding of vegetarian or vegan dog food. It is noteworthy that this is the oldest study, with the smallest sample size, and study conditions furthest removed from the lifestyles of dogs within normal households. Yamada et al. [58] recorded an increase in anaemia following exercise in dogs fed vegetable protein-based diets. This study is further discussed under ‘Specific disorders’.

In 2023 Domínguez-Oliva et al. [59] published a systematic review analysing the impacts of vegan diets on cat and dog health. Included within the 16 studies reviewed were 11 studies of dogs. Collectively considering these studies, they concluded that “there was no overwhelming evidence of adverse effects arising from use of these diets and there was some evidence of benefits. … Much of these data were acquired from guardians via survey-type studies, but these can be subject to selection biases, as well as subjectivity around the outcomes. However, these beneficial findings were relatively consistent across several studies and should, therefore, not be disregarded.”

### 4.3 Specific health disorders (22)

The ten most prevalent health disorders or affected bodily systems within the 2,054 dogs we studied, were: gastrointestinal (e.g., diarrhoea, vomiting), skin/coat, musculoskeletal (muscle or bone), ears, mobility problems, dental/oral (teeth/mouth), anal glands, body weight, eyes and cancer/tumours (Table 9). As noted previously [7], these results are consistent with prior studies. Among 22,333 UK dogs studied in 2016 by O'Neill et al. [20], the most common disorder groups were dental, skin, enteropathy and musculoskeletal. The most common individual disorders were periodontal disease, otitis externa, obesity, overgrown nails and anal sac impaction. Similarly, a 2009–2013 study of 3,884 English dogs found the most common disorders to be otitis externa, periodontal disease, anal sac impaction, overgrown nails and degenerative joint disease [19]. Analyses of Swedish pet insurance records found that skin and gastrointestinal disorders were among the most common [16,17]. And Freeman et al. [18] used a telephone survey to determine that the most common disorders among US dogs were musculoskeletal, dental, and gastrointestinal tract or hepatic disease.

Results from these previous studies were broadly consistent with each other and with results from our 2,054 dogs. However, we found that dental/oral and obesity disorders were lower in the ‘top 10’ rankings within our sample. This appeared even more significant given that our sample included many more neutered animals (77 % vs 45 %) than reported by O'Neill et al. [20], yet obesity and dental disorders are more prevalent among neutered animals (O'Neill et al. [20]. Although dental disease and obesity are not well identified by pet guardians, we sought to minimise the effect of this by relying on reported opinions of veterinarians.

In some cases, difference between our results and other studies may be due to changing prevalences of some diseases, or to differing answer options provided within surveys. For example, unlike O'Neill et al. [20] we did not offer ‘overgrown nails’ as an answer, although respondents were still able to identify musculoskeletal, mobility, or ‘other’ problems, and to provide additional information concerning the latter.

Within our study sample the prevalence of the 22 specific disorders we examined was highest in conventional meat-based dogs for 11 disorders, in raw meat-based dogs for eight disorders, and in dogs fed vegan diets, for three disorders (Fig. 12). After controlling for differences in canine demographic factors via regression models, multiple statistically significant differences were observed between diet groups (Table 10, Table 11). Compared to dogs fed conventional meat, those fed vegan diets had significantly lower odds of suffering from six disorders. These included odds reductions of 61 % for body weight disorders, 56 % for ear disorders, 53 % for musculoskeletal (muscle or bone) disorders, 52 % for gastrointestinal disorders (e.g., diarrhoea, vomiting), 52 % for anal gland problems, and 50 % for dental/oral problems.

When compared to dogs fed raw meat, dogs fed vegan diets had significantly lower odds of suffering from two disorders (Table 10, Table 11). They had odds reductions of 60 % for ear disorders, and of 50 % for musculoskeletal problems. Hence, the significant reduction in risks of suffering from ear and musculoskeletal disorders for dogs fed vegan diets, was consistent when compared to both meat-based diets.

On the other hand, dogs fed vegan diets had significantly increased odds of suffering from internal parasites: 538 % compared to dogs fed conventional meat, and 1,018 % compared to dogs fed raw meat (although the prevalence among dogs fed vegan diets was still only 3 %, or 9/279 affected dogs; Table 6).

For many of the 22 health disorders studied, the number of affected dogs was small (Table 6). This was especially true for dogs fed vegan diets (the smallest dietary group), with less than 10 such dogs affected for 77 % (17) of these 22 disorders. Such small numbers may have partially prevented the detection of additional statistically significant differences. Nevertheless, results within our sample were noteworthy for several additional disorders, in which disorder risks more than halved or doubled (Table 10). No dogs fed vegan diets reportedly suffered from allergies (Table 6). When compared to dogs fed conventional meat, dogs fed vegan diets within our sample also had odds reductions of 66 % for epilepsy, 62 % for behavioural problems and 60 % for eye disorders. Compared to dogs fed raw meat, those fed vegan diets also had odds reductions of 65 % for epilepsy, 61 % for other medical disorders, 59 % for eye disorders, 54 % for respiratory tract disorders and 51 % for injuries. However, compared to dogs fed raw meat, those fed vegan diets also had odds increases of 164 % for behavioural disorders (although the prevalence among dogs fed vegan diets was still only 1 %, or 4/279 affected dogs; Table 6). None of these differences were statistically significant (Table 10).

As noted previously [7], certain of these disorders are related. Considering body weight problems, 85 % of affected dogs were reportedly overweight, and overweight dogs are more likely to have musculoskeletal disorders [48] (p. 783). Dogs suffering from allergies are more likely to have ear disorders [48] (p. 525). All of these specific disorders appeared less common in those dogs fed vegan diets.

In some cases, dietary aetiological causes may explain these results. Diet is an important source of allergens in dogs, and vegan diets lack animal-sourced allergens, notably beef, chicken, fish, pork, lamb [48] (p. 526). No obvious aetiological explanation is available on some other cases, such as the apparently increased odds of internal parasites when vegan diets were fed, or the apparently decreased odds of behavioural disorders in dogs fed raw meat diets, when compared to dogs fed conventional meat-based diets (Table 11). Perhaps some guardians feeding vegan diets incorrectly assume that lack of meat eliminates risks of acquiring internal parasites, and hence use endoparasiticidal treatments less frequently than recommended. Dietary meat is an important internal parasite source – especially when raw meat is fed, but such parasites may also be acquired from the environment or other pets [48]. Hence guardians feeding vegan diets should still administer endoparasiticidal treatments in accordance with veterinary advice. Certain appetitive behaviours may also be increased in dogs fed raw meat, compared to those fed conventional meat-based diets [13]. Perhaps other behaviours, including behavioural disorders, could also be affected, although we are not aware of any evidence to support this.

#### 4.3.1 Consistency with prior related studies: specific health disorders

As mentioned, since we conducted our 2020 survey, key new studies have been published, which also demonstrate reduced levels of specific health disorders, in dogs fed vegan diets. After analysing dietary information from 1,189 dog guardians, including 357 feeding solely vegan diets for three years on average, and also using regression analyses to control for age, sex, neutering status and breed type, Dodd et al. [51] found that dogs fed vegan diets were reportedly less likely to suffer from ocular, gastrointestinal and hepatic (liver) disorders. After analysing dietary information from 100 dog guardians, Davies [52] found that switching to a nutritionally complete vegan dog food resulted in a range of clear benefits after 3–12 months. These included improvements in: coat glossiness, dandruff and erythema (skin inflammation), itchiness (scratching; pruritus), external ear canal crusting (otitis externa), faecal consistency, defaecation frequency, flatus frequency and antisocial smell, anxiety, aggressive behaviour and coprophagia (stool consumption).

Our results also concur with some of those obtained by Kiemer [54] who surveyed 250 dog guardians. As noted, 84.8 % used vegan dog food, 14.0 % used variations of vegetarian diets, and 1.2 % used meat-based commercial dog food. Subgroups provided information about various health changes experienced after switching to a plant-based diet. Overall health was reportedly improved by 33.2 % (and decreased by 0.8 %) of 143 guardians, with activity level reportedly increased by 20.4 % (and decreased by 0.8 %) of 146 guardians. Reductions in dermatological problems were reported by 26.0 % (with increases by 1.2 %) of 135 guardians. Healthier and shinier coats were respectively reported by 34.4 % and 36.8 % of 149 guardians, with less healthy or shiny coats reported by 2.0 %, for each condition. Unpleasant dog odour was reportedly reduced by 31.6 % (and increased, by 0.8 %) of 141 guardians. Dental health was reportedly improved by 19.2 % (and worsened, by 3.6 %) of 142 guardians. Gum health was reportedly improved by 12.8 % (and worsened, by 0.8 %) of 141 guardians. Decreased malodorous breath was reported by 20.8 % (with increases reported by 6.0 %) of 134 guardians.

As noted under ‘Consistency with prior related studies: general health’, Kiemer also reported blood nutrient levels in 20 dogs who were fed a vegan diet for an average of 2.15 years. All 20 dogs of these dogs also had ideal body weights, and all but one had a healthy skin and coat.

The decreased rates of some specific disorders that we observed also concur with Semp's 2014 [55] study. She surveyed 174 vegan dog and 59 vegan cat guardians about the health status of their animals. Thirty-eight reported healthier and shinier coats after changing to vegan diets. Sixteen also described improved odours of their pets. Dermatological problems reportedly resolved in some animals. None of the dogs fed vegan diets that we studied suffered from allergies, which often manifest as skin conditions in dogs [48] (pp. 525–526). Among the dogs we studied, probabilities of suffering from a skin/coat condition varied with diet: 7 % (conventional meat), 8 % (raw meat), and 6 % (vegan diet) (Table 9).

Some of the guardians surveyed by Semp also reported improved stool consistency. This was consistent with the reports of significantly lowered rates of gastrointestinal problems within the dogs fed vegan diets we studied. Depending on diet, these probabilities the prevalence of gastrointestinal problems was: 11 % (conventional meat), 6 % (raw meat), and 5 % (vegan diet) (Table 6).

As mentioned, one study published to date does not support the feeding of vegetarian or vegan dog food. Yamada et al. [58] conducted research on eight dogs, divided into two groups fed animal or vegetable protein-based (VP) diets. It was not clear whether the latter was a vegan diet. Protein comprised around 30 % of each diet. The other macronutrients and energy contents were aligned. The VP-based dogs developed anaemia following exercise. Yamada et al. suggested this could be due to changes in circulating lipid levels (reduction of free cholesterol), resulting in lowered resistance of erythrocytes to haemolysis. However, in this case the experimental regime was very severe. After six weeks of rest dogs were forced to run at 12 km/h for 4 h daily, for two weeks. However, these study results cannot be reliably extrapolated to domesticated dogs, as in this case the exercise regime was very different from that of normal dogs, and the sample size was also too small for reliable extrapolation.

Overall, our results are largely consistent with other studies in this field. Common benefits associated with the feeding of vegan dog food appear to be reductions in the prevalence of body weight and musculoskeletal disorders, allergies, skin, ear and gastrointestinal disorders, and some behavioural disorders. These have all been reported in multiple studies. By 2024, no health disorder had been reported to be more common in dogs fed vegan diets, in more than one study.

### 4.4 Study limitations and further research suggestions

We acknowledge that our study had several limitations, as noted previously [7]. First, dogs were not exclusively fed the study diets. Guardians were asked to “consider the main ingredients within your pet's normal diet,” enabling us to classify them as meat-based or vegan. However, of the 2,536 studied dogs, 76 % also received a variety of treats once or more per day, and 37 % regularly received dietary supplements. Hence, our results may not exactly reflect controlled trials in which diets are exclusively fed, as might occur within a research institute. Instead, they indicate health outcomes following the use of vegan and meat-based diets within normal households, with normal feeding regimes.

One source of potential error was reliance on both quantitative information and opinions supplied by dog guardians. In contrast, large-scale, prospective studies, analysing objective assessments of unambiguous data, are more reliable. More reliable data could be sourced from veterinary clinical examinations, and especially from laboratory assays of physiological indicators within blood and urine. However, to ensure results can be extrapolated to larger dog populations with statistical validity, large animal numbers are required. Unfortunately, this makes such studies costly. These costs were well in excess of our limited research budget. Accordingly, we were forced to rely on guardian-reported data and opinions. This introduced possible error, for example, due to memory lapses. Most at risk of this, were those 5 % (119/2,536) of respondents whose animals were using a therapeutic diet. They were asked to “answer all questions about your animal and their diet, using the 12 months prior to starting their therapeutic or prescription (i.e., medical) diet.” These key instructions were highlighted, and respondents were also advised, “If you cannot recall details, please provide your best estimates, or answer 'unsure' etc. as appropriate.”

Unconscious bias is another source of potential error. This could occur if a guardian expected a better health outcome following their choice to feed a certain diet. Such expectations may exert subtle effects on outcomes assessment, such as assessment of illness indicators, with assessors normally unaware of such effects. This could occur if, for example, a guardian was first asked to report their use of an unconventional diet. Such a respondent could subsequently be more likely to under-report health problems. To minimise such unconscious bias effects, we ensured that survey questions asking about illness indicators were positioned prior to questions about diets. We also took care to ensure that no bias for or against any particular diet type was inherent within survey questions, explanatory text or advertising materials. Whilst such steps minimise rather than eliminate unconscious bias effects, we do not consider any remaining effects to have been more likely in one dietary group than another, and consider their effects on our results were likely minimal.

This confidence was further strengthened by a reanalysis of our data by Barrett-Jolley and German [60]. These investigators used both regression analyses and machine learning predictive modelling to assess the correlation of various factors with guardian opinion of illness severity, using two variables they created: ‘any healthcare problem’ and ‘significant illness.’ They concluded that diet has negligible impact on these, i.e., on guardian opinion of illness severity. In contrast, they concluded that certain other factors are strongly correlated, such as dog age, medication use and numbers of veterinary visits. This was predictable: dogs who are old, using medication and have high numbers of veterinary visits, are more likely to be considered by guardians to be unwell or to have illness of greater severity.

We acknowledge that our objectives differed from those of Barrett-Jolley and German. We drew conclusions about which diet was healthiest and least hazardous overall, based on seven general indicators of illness, the reported prevalence of 22 specific healthcare disorders, and also, prior studies indicating hazards (pathogens and nutritional imbalances) associated with raw meat diets. In contrast, Barrett-Jolley and German assessed correlations between guardian opinion of illness severity, and factors such as those above. The data sets examined were also slightly different. Barrett-Jolley and German excluded data from dogs younger than one year (n = 26) and from 111 respondents who were not primary decision-makers with respect to dog diet choice. And although Barrett-Jolley and German similarly controlled for differences in age, sex, neutering status and breed size, they did not control for differences in exercise levels as we did. Nevertheless, Barrett-Jolley and German showed that diet fed has negligible impact on guardian opinion of illness severity, increasing our confidence in the validity of the guardian-reported results we relied on.

Despite these results, and the methodological steps we implemented to minimise potential errors, error is unlikely to be entirely avoidable when guardian-reported data are used. As a further mitigating step, we also asked guardians to report the health assessments of their veterinarians, concerning their animals. These assessments – rather than guardian opinions – were used to determine indicators such as the percentage of unwell animals in each dietary group, the average number of health disorders per unwell animal, and the prevalence of the 22 most common health disorders. To increase the reliability of these reported veterinary assessments, we excluded from this subset all guardians who had not seen veterinarians at least once within the last year, or who felt unsure of their veterinarians' assessments. We also provided guardians with the opportunity to provide their own opinions concerning animal health status, partly in the expectation that knowledge of this opportunity would encourage guardians to more accurately report their veterinarians’ assessments, if they disagreed with these.

Crucially, we did not rely solely on reported opinions and healthcare assessments. We also obtained data on several more objective indicators of illness, including the frequency of veterinary visits, and the use of medications and therapeutic diets. It is unlikely that more than a very small proportion of these data were incorrect, or that any errors were more prevalent in any one dietary group, than another.

Our survey results may also have been affected by the global coronavirus (COVID-19) pandemic. It was made available from May–December 2020, during associated lockdowns. This may have decreased veterinary visits in some countries, and the use of medications or therapeutic diets, notwithstanding partial mitigation of these effects via remote veterinary consultations and prescribing in many regions. However, because these healthcare indicators often signal a health problem, decreases would have made our results more conservative.

It is also true that our respondents were not fully representative of dog guardians. People lacking internet access would have been unable to complete this survey. Males were also underrepresented, comprising only 7 % of respondents. Our respondents were not evenly distributed geographically, being primarily located in the UK (71 %) or Europe (15 %). However, we see no reason why such anomalies would have appreciably altered reported data or opinions concerning indicators of illness.

Finally, although sufficient to draw conclusions concerning the overall health of dogs maintained on the three diets studied, our participant numbers may not have been sufficient to detect statistically significant differences in risks of specific medical disorders for disorders with very low prevalences. Numerous disorders fell within this group (Table 6). Significantly larger numbers might allow detection of such differences. Larger sample sizes might also allow controlling for possible effects of additional canine demographic factors such as body condition and weight, specific breed, exercise levels, social factors or season/weather. Health consequences of smaller dietary groups, such as vegetarian animals, and of new diets such those based on yeast or *in vitro* meat products, could also be investigated. Ensuring sufficiency of sample sizes to allow detection of statistically significant differences between subgroups could require focusing exclusively on such interest groups.

As noted previously [7], results of greater reliability could also be obtained from large-scale cross-sectional, or ideally, prospective longitudinal studies of dogs maintained on different diets. Ideally, these should utilise more objective data, such as veterinary clinical examination findings, veterinary medical case histories, and laboratory data. Significantly greater research funding and resources would need to be secured for a large-scale study of this kind.

### 4.5 Safeguarding health

Our results and those of the other studies described, indicate that the healthiest diets for dogs, among vegan, conventional meat and raw meat-based diets, are nutritionally-sound vegan diets. However, as noted previously [7], all dietary choices may prove hazardous, if not formulated to be nutritionally-sound, or if contaminated by pathogens or other hazards. The latter hazards are most common in raw meat diets, which have been repeatedly demonstrated to have nutritional deficiencies, such as specific vitamin deficiencies and calcium/phosphorous imbalances [37,42], as well as bacterial and non-bacterial pathogens and zoonoses, risking the health of both dogs and their guardians [[38], [39], [40],[44], [45], [46]]. Because of such hazards, as well as their inferior health outcomes when compared to nutritionally-sound vegan diets, raw meat diets are not normally recommended by veterinarians, nor by us.

Even when feeding other diets, guardians should take care to ensure diets are nutritionally complete and balanced by checking labelling claims of nutritional adequacy. Guardians are also advised to check company information about steps taken to ensure nutritional soundness and consistency of diets [12]. It may also be appropriate to consider life stage (e.g., young, old) and physiological status (e.g., pregnant, heavily exercising).

## 5 Conclusions

Pet guardians and pet food companies are increasingly concerned about the environmental sustainability and ‘food animal’ welfare impacts of meat-based pet food [2,3,61]. Driven by consumer demand, companies are developing a range of alternative diets to address these concerns. Among these, vegan pet foods are the most developed. When considering whether to choose alternative diets, health outcomes are the leading consumer concern [7]. Yet, by 2021, studies of health outcomes comparing dogs fed vegan and meat-based diets were few, and often limited in size.

Our 2022 study of 2,639 dogs and their guardians was one of the first to examine such variations in health outcomes. After studying dogs fed conventional meat (1,370 = 54 %), raw meat (830 = 33 %) or vegan (336 = 13 %) diets for at least one year, we found that dogs in the latter two groups had significantly better health outcomes than dogs fed conventional meat-based diets. However, differences in average ages were sizeable and statistically significant, with dogs fed raw meat being younger, and those fed vegan diets being older, on average, than dogs fed conventional diets [7]. Lower ages can improve health outcomes.

Accordingly, this follow-up study controlled for differences between dietary groups in medically-relevant canine demographic variables, including age, sex, neutering status, breed size and unusually high exercise levels. After applying this to seven general indicators of illness, and 22 specific disorders, dogs fed vegan diets had better health outcomes than dogs fed either raw or conventional meat-based diets (Table 12). These results are summarised in Fig. 13. This trend was clear and consistent, with lowered odds of illness indicators usually both substantial and statistically significant, for dogs fed vegan diets (Table 2, Table 3). Dogs reportedly considered by veterinarians to be unwell comprised 49 % of those fed conventional meat, 43 % of those fed raw meat, and 36 % of those fed vegan diets (Table 7).

When considering the prevalence 22 of the most common canine health disorders, within our sample the probabilities of suffering from a disorder respectively appeared highest in conventional meat-based dogs (for 11 disorders), raw meat-based dogs (for eight disorders), and vegan dogs (for three disorders) (Fig. 12). Dogs fed vegan diets had significantly lower odds of suffering from multiple health disorders (six, compared to dogs fed conventional meat, and two, compared to dogs fed raw meat). Dogs fed vegan diets had significantly greater odds of suffering from only a single health disorder, when compared to dogs fed conventional meat, and also only a single (identical) disorder, when compared to dogs fed raw meat (Table 11). Overall, the number of dogs suffering from most health disorders was very small (Table 6). This may have prevented the detection of additional statistically significant differences between diet groups in the prevalence of certain health disorders.

Additionally, numerous studies have demonstrated nutritional deficiencies or imbalances, and pathogen hazards, associated with raw meat diets [[37], [38], [39], [40], [41], [42], [43], [44], [45], [46]]. Accordingly, when collectively considering the evidence concerning conventional meat, raw meat and vegan diets, from our study, and from others in this field, the healthiest and least hazardous diets for dogs are nutritionally sound vegan diets. However, to safeguard health, diets of all kinds should always be formulated to be nutritionally complete and balanced, and manufactured in accordance with best practice standards [9].

## Ethics declaration

This study was reviewed and deemed exempt from ethics approval by the RKE Ethics Committee, University of Winchester, UK, with the reference number: RKEEC200304_Knight, dated March 4, 2020. All participants were informed that consent to participate in the study and publish their data would be assumed on completion and submission of the study questionnaire.

## Funding

This research was partly funded by food awareness organisation ProVeg International (www.proveg.com). AK received this award ID: Oct2019-0000000286. Funding for open access publication was sourced from Representing Animals. However, these funders played no role in study conceptualisation, design, data collection and analysis, preparation of the resultant manuscript nor decisions relating to publication.

## Data availability statement

Our data analysed, along with the R code used for its statistical analysis, are accessible at https://osf.io/nbepu.

## CRediT authorship contribution statement

**Andrew Knight:** Writing – review & editing, Writing – original draft, Visualization, Validation, Supervision, Software, Resources, Project administration, Methodology, Investigation, Funding acquisition, Formal analysis, Data curation, Conceptualization. **Alexander Bauer:** Writing – review & editing, Writing – original draft, Visualization, Validation, Software, Methodology, Formal analysis, Data curation, Conceptualization. **Hazel J. Brown:** Writing – review & editing, Supervision.

## Declaration of competing interest

The authors declare the following financial interests/personal relationships which may be considered as potential competing interests:Andrew Knight reports financial support was provided by Representing Animals. Andrew Knight reports financial support was provided by ProVeg International. Andrew Knight reports a relationship with Food System Research Fund that includes: funding grants. Andrew Knight reports a relationship with Edgard & Cooper that includes: funding grants. Alexander Bauer reports a relationship with Representing Animals that includes: consulting or advisory. Alexander Bauer reports a relationship with VEGDOG that includes: consulting or advisory. If there are other authors, they declare that they have no known competing financial interests or personal relationships that could have appeared to influence the work reported in this paper.

## Figures and Tables

**Fig. A2:**
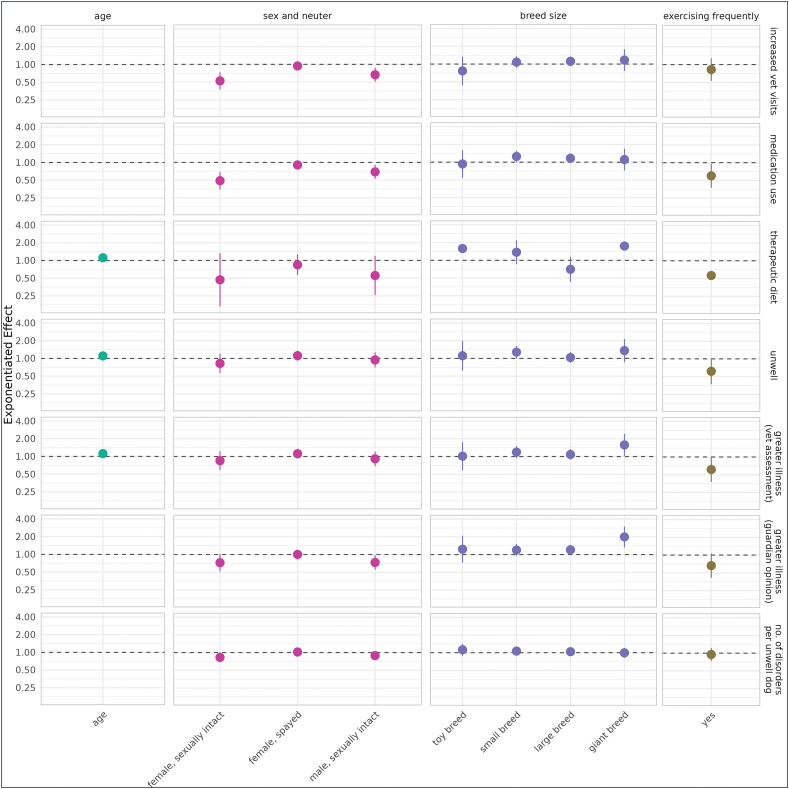
Visualisation of all control variables effect estimates, on a logarithmic y-axis, for all seven regression models among 2,536 dogs fed three main diets, as listed in Table 1. Note: Dog numbers in some groups were lower than 2,536, as described under Results. Dots mark the exponentiated effects, with bars corresponding to exponentiated 95 % confidence intervals. As noted in Table 1, the reference categories for variables ‘sex and neuter’, ‘breed size’ and ‘exercise level’ are ‘male, castrated’, ‘medium size’ and ‘exercise not unusually high’, respectively. The effects of the other categories indicate average differences compared to these reference categories. For models with a nonlinear age effect the respective panel is left blank. These nonlinear age effects are visualised in Figures A3 – A6.

**Table A1:**
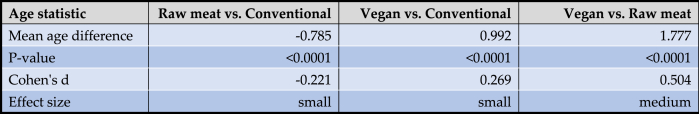
Age differences between 2,534 dogs fed three main diets.

**Table A2:**
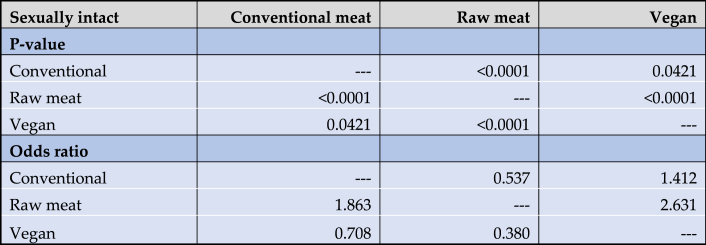
Differences in odds of being sexually intact, between different diets, for 2,536 dogs fed three main diets. After Knight et al. [7].

**Table A3:**
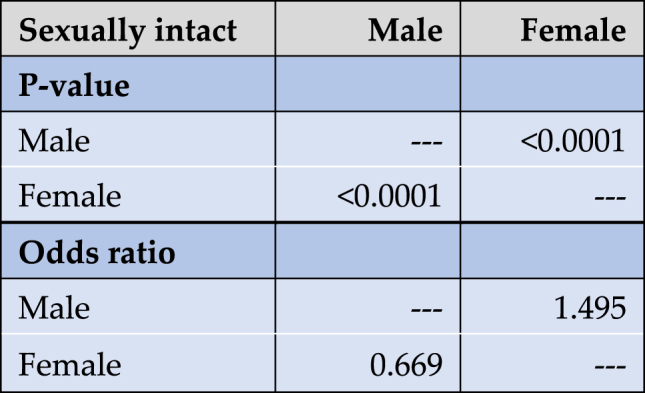
Differences in odds of being sexually intact, between males and females, for 2,536 dogs fed three main diets. After Knight et al. [7].

**Table A4:**
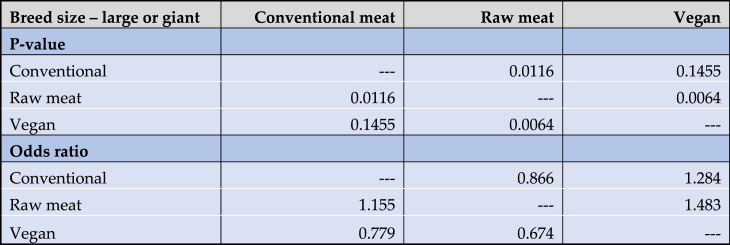
Differences in odds of being a large or giant breed, compared to a toy, small or medium breed, between different diets, for 2,536 dogs fed three main diets.

**Table A5:**
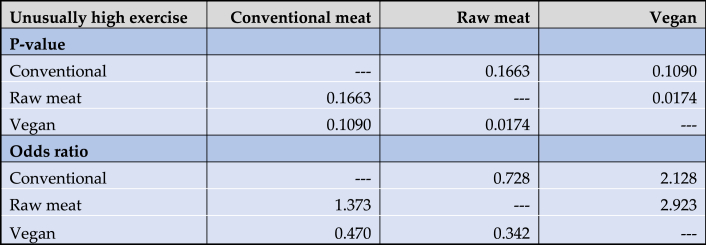
Differences in odds of exercising at unusually high levels, between different diets, for 2,530 dogs fed three main diets.

Note: Unusually high exercise levels were exemplified by racing greyhounds, working farm dogs, or police dogs.

## Appendix A Survey parts

Steps included within our 2020 survey of 2,526 dog (and 1,369 cat) guardians.

## Appendix B Effects of control variables

The following figures visualise the estimated effects of all control variables, in all seven regression models used for the general indicators of illness.
